# Modular Structure and Polymerization Status of GABA_A_ Receptors Illustrated with EM Analysis and AlphaFold2 Prediction

**DOI:** 10.3390/ijms251810142

**Published:** 2024-09-21

**Authors:** Chloe Kan, Ata Ullah, Shangyu Dang, Hong Xue

**Affiliations:** Division of Life Science, Hong Kong University of Science and Technology, Clear Water Bay, Hong Kong, China; ckanab@connect.ust.hk (C.K.); aullah@connect.ust.hk (A.U.); sdang@ust.hk (S.D.)

**Keywords:** α1β2γ2 Type-A γ-aminobutyric acid (GABA_A_) receptor, pentameric structure, homopentamers, AlphaFold2, ligand-gated ion channel, Cys-loop receptors

## Abstract

Type-A γ-aminobutyric acid (GABA_A_) receptors are channel proteins crucial to mediating neuronal balance in the central nervous system (CNS). The structure of GABA_A_ receptors allows for multiple binding sites and is key to drug development. Yet the formation mechanism of the receptor’s distinctive pentameric structure is still unknown. This study aims to investigate the role of three predominant subunits of the human GABA_A_ receptor in the formation of protein pentamers. Through purifying and refolding the protein fragments of the GABA_A_ receptor α1, β2, and γ2 subunits, the particle structures were visualised with negative staining electron microscopy (EM). To aid the analysis, AlphaFold2 was used to compare the structures. Results show that α1 and β2 subunit fragments successfully formed homo-oligomers, particularly homopentameric structures, while the predominant heteropentameric GABA_A_ receptor was also replicated through the combination of the three subunits. However, homopentameric structures were not observed with the γ2 subunit proteins. A comparison of the AlphaFold2 predictions and the previously obtained cryo-EM structures presents new insights into the subunits’ modular structure and polymerization status. By performing experimental and computational studies, a deeper understanding of the complex structure of GABA_A_ receptors is provided. Hopefully, this study can pave the way to developing novel therapeutics for neuropsychiatric diseases.

## 1. Introduction

Neurotransmitters function to transmit signals from nerve cells to other target cells through synapses and are important for maintaining neuronal balance. Inhibitory neurotransmitters reduce the chances of an action potential and interact with corresponding receptors to prevent over-excitation. γ-aminobutyric acid (GABA) functions as the major inhibitory neurotransmitter in the human central nervous system, and its main corresponding receptor is the type-A γ-aminobutyric acid (GABA_A_) receptor [1,2,3]. When GABA binds to its receptor, the GABA_A_ receptors undergo conformational changes, leading to an influx of negatively charged chloride ions and terminating an action potential by causing hyperpolarization [3,4].

GABA_A_ receptors are part of the superfamily of Cys-loop pentameric ligand-gated ion channels (pLGICs), which include other neurotransmitter receptors, such as the glycine receptor (GlyR) [5]. GlyRs are closely related to GABA_A_ receptors [6,7]. Both receptors possess the ability to form heteropentamers with different subunits, while several specific subunits can also form homopentamers [8]. Currently, 19 subunits of the GABA_A_ receptor have been discovered in humans, namely the α1–6, β1–3, γ1–3, δ, ε, θ, π, and ρ1–3 subunits [9,10]. The structure of each subunit usually includes a hydrophilic extracellular domain with the Cys-loop and four hydrophobic transmembrane domains [10,11]. Various combinations of subunits may be assembled to construct the receptors. Still, the most common form consists of two α, two β, and one γ subunits, with the most abundant subtype being α1β2γ2 in a 2:2:1 ratio, accounting for 43% of all GABA_A_ receptors in the brain [1,12,13]. In this predominant subtype of GABA_A_ receptors, alternating α1 and β2 subunits are connected by a single γ2 subunit. Apart from the GABA binding site, the GABA_A_ receptors also have a benzodiazepine (BZ) drug binding site. The GABA binding site is located between the α and β subunits, while the BZ binding site is located between the α and γ subunits [14,15]. Dysfunction of the GABA_A_ receptors can lead to neuropsychiatric disorders [16], which cause the GABA_A_ receptors to play an important role in the study of receptor-based drugs, such as BZs. To design specific drugs and study the ligand binding mechanism of the receptors, there is an urgent need to identify the atomic structure of the receptor.

Ligand binding sites in the GABA_A_ receptor are widely studied. The α1 subunit is the most highly expressed GABA_A_ receptor subunit [17,18,19], and some of its residues have been identified to interact with GABA agonists [20,21,22,23]. It is well known that the aromatic amino acids—phenylalanine (Phe), tyrosine (Tyr), and tryptophan (Trp), are frequently involved in π interactions, which often play an important role in protein structural formation and ligand binding [24,25]. Indeed, GABA agonist binding sites are lined with aromatic residues, including Phe200 and Tyr205 on the β subunit and Phe46 and Phe65 on the α subunit [26]. Interestingly, Phe65 (Phe64 on bovine homolog [27]) is conserved among the six α subunits as well as the γ2 subunit and is one of the residues that have been indicated to interact with the GABA agonist, muscimol [20,28]. Amongst the 20 amino acids, Trp is the largest and contains two aromatic rings. Perhaps due to its complexity, Trp is the least abundant amino acid and the most energy-consuming [29,30]. It has been reported to play a role in stabilizing the structures of membrane proteins [31,32,33]. In fact, the invariant residues Trp69 and Trp94 in the α1 subunit have been identified as crucial residues for the GABA_A_ receptor pentameric assembly [34]. Previous studies have suggested various roles in these two residues associated with channel opening [35] and the folding of loops [36], leading to potentially altering conformational characteristics. One study performed a point mutation on these residues, expressing the α1 peptide with β2 and γ2 subunits. The results demonstrated that the mutant α1 peptide did not form pentamers with β2 and γ2 subunits and could not bind to the BZ ligand, [^3^H]flunitrazepam [34]. Thus, aromatic residues in the α1 subunit may play a role in the GABA_A_ receptor pentamer formation and ligand binding mechanisms.

Incorporating an additional β2 subunit in the GABA_A_ receptor has been proposed to increase the binding sensitivity of GABA [37], suggesting an intriguing role of the β2 subunit in GABA binding and structural formation. Again, mutations in the aromatic residues Tyr97, Tyr157, and Tyr205 on the β2 subunit greatly reduced the binding rate of GABA [38]. Similarly, important domains have been investigated within the γ2 subunit, for example, a region for flunitrazepam binding that consisted of Tyr-58 as an essential residue for high-affinity binding [39]. Hence, evidence has highlighted the significance of aromatic residues in ligand binding in the most predominant form of the α1β2γ2 GABA_A_ receptors. These studies surely aid in developing pharmacological drugs targeting the GABA_A_ receptor and give insight into the binding mechanism of ligands. Still, structural studies are needed to explore potential conformational changes and intermolecular interactions with ligands.

Previously, researchers overexpressed the major subunits of the GABA_A_ receptor in *E. coli* to extract structural information and identify key binding residues [40,41,42]. Subsequently, the Cys139–Leu269 fragment of the α1 subunit was successfully expressed, resulting in the formation of rosette-like structures [41,42,43]. Methods such as ala-scanning and base substitution revealed several important residues. In particular, five conserved Cys and Trp residues were identified in the membrane-proximal β-rich (MPB) domain and associated with the structural stability of an “immunoglobulin-like” (Ig-like) fold of the α1 subunit [43]. For instance, in some Ig-like proteins with two packed β-sheets, an additional Trp residue is packed against the cysteine disulphide bridge between the β-sheets [44]. Similarly, Trp residues located in proximity to the Cys-loop were shown to affect the structural stability of the Cys-loop receptors [43,45]. This provides invaluable insights into the secondary structure of the GABA_A_ receptor subunits and the potential motifs in identifying Ig-like domains in Cys-loop receptors.

High-resolution atomic structures of the GABA_A_ receptor have been determined with the aid of technological advancements and methods such as 3D classification, negative staining microscopy, and cryo-EM. The earliest high-resolution cryo-EM structures of the α1β2γ2 GABA_A_ receptor were successfully determined in 2018 (PDB IDs: 6D6U, 6D6T, 6DW0, 6DW1) [46,47], providing an additional foundation for investigating the receptor’s architecture and ligand binding. Yet some structures described in the studies were unusual [48], particularly in one conformation where the γ2 subunit collapsed into the pore of the receptor [46]. Another cryo-EM structure was solved using full-length subunits of the α1β3γ2 receptor subtype, but the membrane-spanning segments of the M3–M4 loops were reported to be disordered [15]. A majority of the studies focus on the hetero-pentameric receptor, in which many include ligands in complex with the receptor (e.g., PDB ID: 6X3X, 6X3Z, 8DD2, 8DD3, 8G4N, 8SGO, 8VQY, 8VRN) [49,50,51,52,53,54]. Interestingly, one particular study presented the first 3D crystal structure of a truncated β3 subunit homo-pentamer (PDB ID: 4COF) [55], encouraging more researchers to explore the possibility of the homo-pentameric structures of the GABA_A_ receptor subunits. These cryo-EM structures can provide an invaluable source of information on the interactions at the subunit interfaces.

As protein research advances, there has been a rising trend in utilizing data-driven approaches to aid analysis and predict protein structures. Novel computational approaches have emerged in recent years due to the increasing focus on bioinformatics and artificial intelligence (AI). It is not surprising that AI and machine learning have been used to develop a model for the prediction of 3D protein structures. AlphaFold [56] is an open-source neural network-based method for predicting protein structures with atomic accuracy. By using multi-sequence alignment and the available knowledge about the target protein, AlphaFold can create a detailed visual model of the structure [57]. This creates an opportunity to compare the similarities and differences between the prediction models and experimental cryo-EM structures for a more robust atomic model. Indeed, recent studies have combined cryo-EM data and AlphaFold predictions in hopes of obtaining a more complete model of protein structures [58,59,60]. Analysing the similarities and differences between the experimental and predicted models may provide new insights and contribute to a better understanding of many complex and fuzzy protein structures, such as the GABA_A_ receptor.

Constant improvements in GABA_A_ receptor models are needed to identify the binding mechanisms of ligands, understanding the interactions between subunits, and explaining the assembly pathway of the receptor. However, exploring eukaryotic membrane proteins is challenging due to their functions depending on their native environment. Therefore, using previous studies as a reference [40,41], the present study utilises truncated fragments of the α1, β2, and γ2 subunit protein samples, aiming for a higher-resolution structure of the key extracellular residues of GABA_A_ receptors. Although GABA_A_ receptors may consist of various subunits, the most abundant form consists of α1, β2, and γ2 only. However, the formation mechanism of the predominant GABA_A_ receptor is still unknown, such as intermediate structures and how different subunits interact with each other freely within their native environment. This study aims to utilize recombinant α1, β2, and γ2 subunit fragments, negative staining EM, and neural network-based model AlphaFold2 [56] for elucidating the structures of GABA_A_ receptors and comparing them to existing cryo-EM structures. By investigating the pentameric formation mechanism of the GABA_A_ receptor’s three major subunits, this study will hopefully increase our understanding of the mechanism of complex protein structures and potentially pave the way to the development of novel therapeutics for neuropsychiatric diseases.

## 2. Results

### 2.1. Amino Acid Sequence Alignment

The amino acid sequence of the α1, β2, and γ2 subunits have previously been identified. Each subunit consists of a long extracellular N-terminus containing a Cys-loop, four transmembrane domains, an intracellular loop, and a short C-terminus [2]. The ligand binding sites of GABA and BZ are located in the receptor’s extracellular domain [46,61,62]. Due to the complication of expressing and purifying whole protein subunits, extracellular domains involving most of the binding sites on the GABA_A_ receptors were expressed and purified. The selected domains were Gln28–Arg248 in the α1 subunit (25 kDa), Gln25–Gly243 in the β2 subunit (25.2kDa), and Gln40–Gly273 in the γ2 subunit (27.5 kDa).

**Figure 1:**
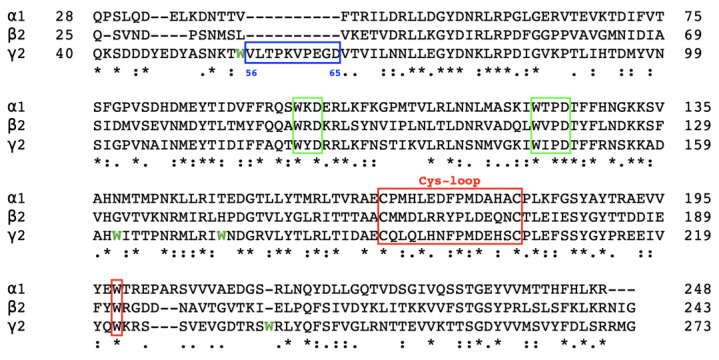
Amino acid sequence alignment for the extracellular domain fragments of human GABA_A_ receptor α1, β2, and γ2 subunits. Alignment by MAFFT in CLUSTAL format. Asterisks (*) show conserved and identical sequences, colon (:) show conservative substitution, and dot (.) shows semi-conservative mutations. Conserved sequences between all three subunits are >26% of the total sequence, as denoted with (*). The additional 10 amino acid sequences in the γ2 fragment, absent in the α1 and β2 fragments, are highlighted in a blue box with the amino acid number denoted below. The WxD and WxPD motifs are shown in a green box. The Cys-loop is highlighted in a red box, and the tryptophan residue within its vicinity is also in red. The extra Trp residues in the γ2 fragment are coloured in green and bolded.

To gain initial insights into the possible structural similarities and differences between the α1, β2, and γ2 subunits of the GABA_A_ receptor, amino acid sequence alignment was performed, as shown in Figure 1. The three subunit fragments of key residues have sequence homology of >26%, with multiple conserved sequences across all three fragments. Genetic analysis indicated that the subunit genes are clustered on chromosome 5, suggesting a possibility of gene duplication of clusters during evolution [2,9,11]. The conserved aromatic residues were particularly noted due to their roles in protein structural formation and ligand binding, especially the least abundant amino acid—tryptophan. Two conserved motifs containing a tryptophan residue (W) were identified—the WxD motif and WxPD motif [45]. Furthermore, a conserved Trp residue was located at 18 residues on the C-terminal side of the second cysteine in the Cys-loop. The percent identity matrix on each subunit (Table 1) indicates that α1 and γ2 subunits have higher sequence homology than with β2. However, there is an additional 10-amino-acid sequence in the γ2 subunit fragment, which may cause differences in the formation of the subunit’s structure. It is also important to note that the γ2 subunit fragment contains four extra Trp residues compared to the α1 and β2 subunit fragments. There are only three Trp residues in both α1 and β2 fragments, which is around 1.36% and 1.37% of the fragments, respectively. However, there are seven Trp residues in the γ2 fragment, which is around 3% of the fragment. This is higher than the average frequency of 1–2% Trp residues in proteins [29,63,64]. Thus, it is natural to speculate that the four extra Trp residues may play a role in creating unique structural properties of the γ2 fragment that vary from the α1 and β2 fragments.

### 2.2. Expression and Purification of α1, β2, and γ2 Subunit Fragments

**Figure 2:**
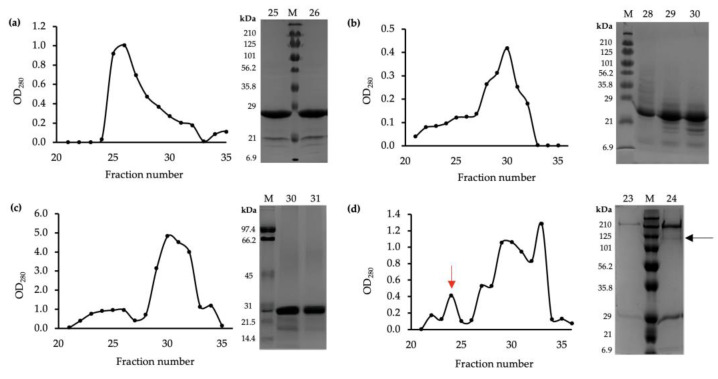
Purification of the protein fragments by gel filtration chromatography. Eluents were collected in numbered Falcon tubes corresponding to the fraction number. The concentration of protein in fraction numbers 21–35 indicated by OD280 measured by NanoDrop. M = marker from Bio-Rad SDS-PAGE Broad Range Standards. Prestained standard was used in (**a**,**b**,**d**), and unstained standard in (**c**) was visualised with Coomassie brilliant blue R-250. A 12% SDS-PAGE was performed for fraction numbers with the highest OD280, representing the highest concentration. (**a**) Concentrations of α1 subunit (Gln28-Arg248 fragment) in consecutive fractions, with eluent numbers 25 and 26 having the highest concentrations. SDS-PAGE visualises the molecular weight to be between 20.6 kDa and 28.9 kDa, corresponding to the 25 kDa size of the fragment. (**b**) Concentration of β2 subunit (Gln25-Gly243 fragment) in consecutive fractions, with eluent numbers 29 and 30 having the highest concentrations. SDS-PAGE visualises the molecular weight to be between 20.6 kDa and 28.9 kDa, corresponding to the 25.2 kDa size of the fragment. (**c**) Concentration of γ2 subunit (Gln40-Gly273 fragment) in consecutive fractions, with eluent numbers 30 and 31 having the highest concentrations. SDS-PAGE visualises the molecular weight to lie slightly below the 28.9 kDa band of the marker, which corresponds to the 27.5 kDa of the fragment. (**d**) Expression and purification analysis of 2α:2β:1γ GABA_A_ receptor complex. Eluent number 23 showed the highest concentration of oligomers (red arrow). Fractions after 25 were considered as concentrations of individual subunits. SDS-PAGE shows the concentration and size of the fragments from eluents number 23 and 24. The black arrow shows the α1β2γ2 GABA_A_ receptor complex with an approximate size of 125 kDa.

After the three fragments were expressed in a prokaryotic vector and purified by washing and precipitation, the solubilized protein pellet was loaded in a Superdex column for gel filtration chromatography and then collected into eluents corresponding to the consecutive fraction numbers. Measurement of optical density at 280 nm (OD_280_) shows protein elution at the absorbing peaks and indicates the concentration of protein obtained. When the purified proteins were applied to the Superdex 200 column, bound proteins were eluted between fraction numbers 20 and 35, as observed in Figure 2. A higher OD_280_ suggests a higher protein concentration [65] thus allowing for dialysis for further structural visualization and negative staining. SDS-PAGE was used to confirm the molecular weight of the purified protein fragments. As shown in Figure 2, the peak of each protein contains a polypeptide band with a molecular mass corresponding to each fragment size. In an attempt to recreate the human heteropentameric α1β2γ2 GABA_A_ receptor, purified proteins were combined in a 2:2:1 ratio of α1, β2, and γ2, respectively. However, the abundance of the protein complex obtained was much less than the individual fragments, as seen in the SDS-PAGE band thickness in Figure 2d.

### 2.3. Visualisation of Protein Structures Using Negative Staining Electron Microscopy (EM)

**Figure 3:**
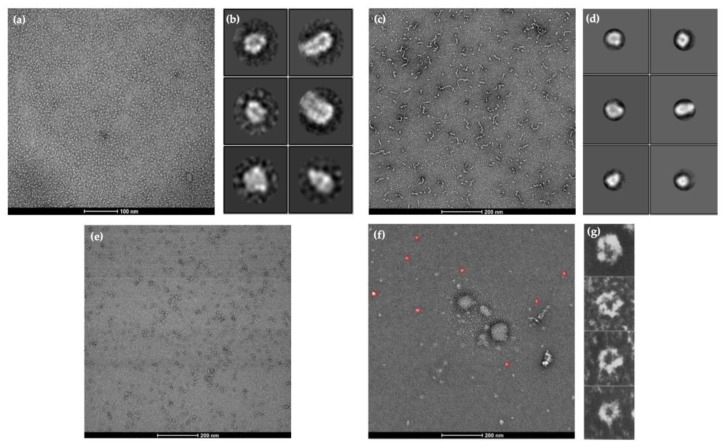
Negative staining and visualization by electron microscopy (EM). Results from formvar carbon-coated copper grids were visualised under EM. Particles were manually picked from 20 micrographs and used to generate 2D averages**.** (**a**) Negative staining of α1 subunit (Gln28-Arg248) and (**b**) 2D classification of α1 homopentamer structures from 2D averages. (**c**) Negative staining of β2 subunit (Gln25-Gly243) and (**d**) 2D classification of Gln25-Gly243 protein fragments from 2D averages suggest a similar pentameric structure. (**e**) Negative staining of γ2 subunit (Gln40-Gly273). (**f**) Negative staining of the 2α:2β:1γ GABA_A_ receptor complex. The protein structures circled red are considered as stable particles, which were subsequently picked and used for (**g**) 2D classification, indicating pentameric structures.

In pursuit of a high-resolution molecular structure, negative staining was performed following the confirmation of molecular weights. Figure 3a depicts α1 subunit negative staining, showing clear protein structure without unusual aggregation. The 2D classification in Figure 3b shows the top views indicating a pentameric shape and the side views suggesting a central cavity. This verifies that α1 subunit fragments successfully formed homopentamers with rosette-like structures and a central cavity as previously described in Xue et al. [41]. Although negative staining and 2D classification of β2 subunit fragments also suggest a homopentameric structure similar to the α1 subunit homopentamer, there seemed to be unusual aggregation, as seen in Figure 3c. Furthermore, the concentration of the β2 subunit fragments was relatively lower than the other fragments obtained (Figure 2b), which could be a reason why the EM images obtained were not of high resolution. On the other hand, the concentration of γ2 subunit fragments obtained was much higher (Figure 2c), but negative staining (Figure 3e) did not identify any pentameric structures for further imaging. In addition to the subunit fragments, the predominant GABA_A_ receptor structure was also analysed.

### 2.4. AlphaFold2 Prediction of α1, β2, and γ2 Homopentameric Structures

The sequence of the fragments obtained from UniProt [66] was applied to AlphaFold2 on ColabFold (Ver. 1.5.5) [57] and tested as homopentamers. For each pentameric structure, a total of 5 models with different structures and confidence levels were created by AlphaFold2, as shown in Supplementary Figure S1. The best model with the highest per residue confidence (pLDDT) was chosen for further analysis in UCSF ChimeraX software (Ver. 1.8) [67,68]. For α1 homopentamer, the confidence score from AlphaFold2 is 86.3 with the predicted template modelling score (pTM) of 0.864, above the 0.75 confidence cut-off (Supplementary Figure S2e). The high concentration and resolution from negative staining, as well as the high structural prediction confidence in AlphaFold2, indicates that the α1 subunit fragment can successfully form homopentameric structures, as expected. Furthermore, the β2 subunit fragment homopentamer structural prediction has a high pLDDT of 91.2 and pTM of 0.9 (Supplementary Figure S2f). The confidence of prediction is even higher than the α1 homopentamer, suggesting a high potential of the β2 subunit forming homopentamers. This is also evident in the negative staining EM results. Similarly, the pentameric GABA_A_ receptor structural prediction had a high pLDDT of 87.8 and pTM of 0.889, supporting its pentameric formation seen in negative staining. Although AlphaFold2 successfully predicted the γ2 subunit pentamer formation with pLDDT = 82.5 and pTM = 0.833, no pentameric structure was seen in the negative staining results (Figure 3e). To further investigate the possible reasons, the secondary structure of the highest-ranked prediction models were analysed in ChimeraX.

**Figure 4:**
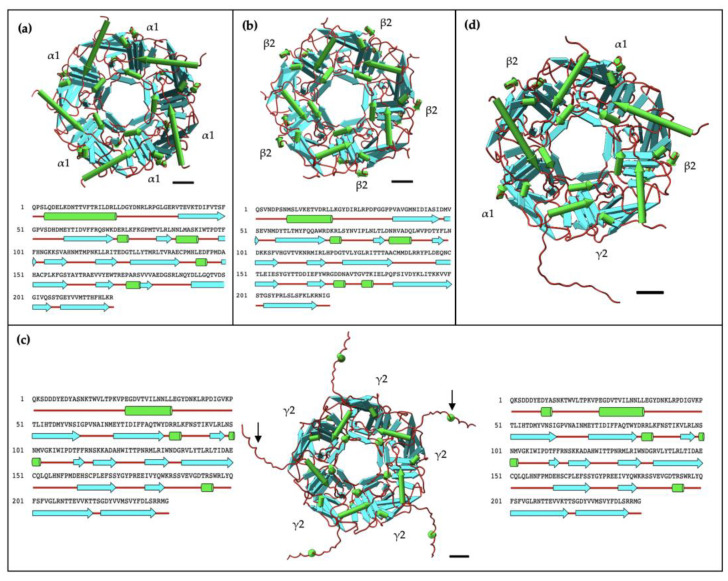
AlphaFold2 predicted structures. Alpha helices are depicted as green “pipes”, beta sheets as cyan “planks”, and red strands as “coils”. The secondary structure is aligned under the amino acid sequence of a single subunit fragment of the corresponding homopentameric structure. The scale bar denotes 10 Å (1 nm). (**a**) α1 subunit fragment, (**b**) β2 subunit fragment, and (**c**) γ2 subunit fragment all show the potential of forming homopentamers based on AlphaFold2 prediction. Black arrows show the two different secondary structures of the individual γ2 subunit fragment, one without the three-residue helix (left) and one with (right). (**d**) The structure of the α1β2γ2 GABA_A_ receptor complex is also predicted with the protruding segment on the γ2 subunit. Pipes and planks are visualised with UCSF Chimera (Ver. 1.17.3).

The α1 homopentamer and β2 homopentamer AlphaFold2 models have similar structures (Figure 4a,b), with a smooth pentameric shape and a clear central cavity. In particular, the central cavity of the β2 seems to be slightly larger than the central cavity in α1 homopentamer. In contrast, the γ2 homopentamer has protruding residues that are not tucked into the pentameric structure, which may be affecting the ability of the fragments to form homopentamers. Interestingly, the AlphaFold2 prediction of a γ2 homopentamer consisted of subunits with slightly different secondary structures. Four of the five γ2 subunits in the homopentamer have a short three-residue helical structure near the beginning of the fragment (Figure 4c). This demonstrates two possible folding methods of the γ2 subunit fragment, which may be difficult to control during protein refolding thus again affecting their ability to form pentamers in vitro. Another notable point from the AlphaFold2 predictions is that the γ2 subunit in the predicted GABA_A_ receptor heteropentamer shows no helical structure in the first 20 residues (Figure 4d). To further investigate the γ2 subunit structure, the four lower-ranked AlphaFold2 models of the γ2 homopentamer were also examined. However, the helical structure was absent in all five γ2 subunits present in the lower-ranked homopentamer models. Thus, despite AlphaFold2’s prediction with the helix as the highest-ranked model, more evidence suggests that the γ2 subunit folding without the three-residue helix is preferred and crucial for forming the predominant human GABA_A_ receptor. Nevertheless, the first twenty-four residues seem to be protruding at similar angles in all the predicted models, therefore the first-ranked model still plays a role in investigating the formation of γ2 homopentamers.

### 2.5. Negative Staining Electron Microscopy (EM) Images and AlphaFold2 Prediction Comparison

**Figure 5:**
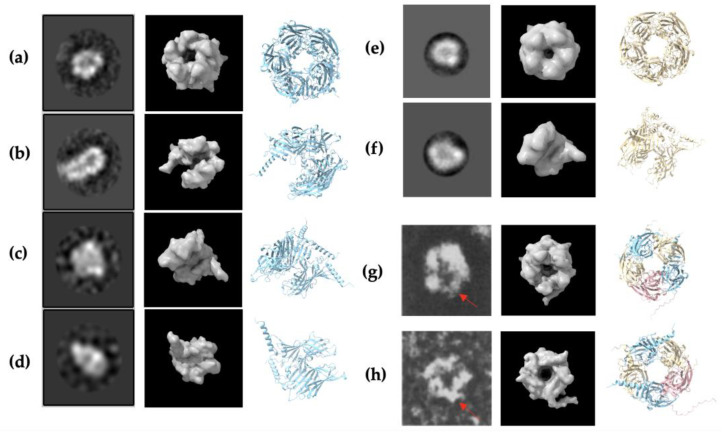
Comparison of negative staining EM images and AlphaFold2 predictions of α1 homopentamer, β2 homopentamer, and GABA_A_ receptor heteropentamer. (**a**–**d**) EM images have a similar shape to molmaps (resolution 10 Å) generated from the AlphaFold2 structure prediction of α1 homopentamer. Structures depicted include (**a**) bottom view of whole pentamer, (**b**) side view with one subunit hidden to mimic the exposed central cavity, (**c**) side view with two subunits hidden, and (**d**) side view with three subunits hidden for a clearer comparison. (**e**,**f**) EM images and molmaps (resolution 15 Å) generated from the AlphaFold2 structure prediction of β2 homopentamer with (**e**) top view of pentamer and (**f**) side view with two subunits hidden to mimic the exposed central cavity. (**g**,**h**) EM images and molmaps (resolution 10 Å) generated from the AlphaFold2 structure prediction of α1β2γ2 GABA_A_ receptor. The α1, β2, and γ2 subunits are shown in blue, yellow and magenta colours respectively, with (**g**) the bottom view of the pentamer, and (**h**) top view of the pentamer. The red arrow identifies the structure resembling the protruding residues of the γ2 subunit. For ribbon structural representations, sky blue fragments represent α1 subunits, yellow represents β2 subunits and pink represents γ2 subunits. Molmaps and AlphaFold2 predictions were visualised with ChimeraX (Ver. 1.8).

To validate the prediction of the AlphaFold2 models, the negative staining electron microscopy (EM) images obtained for α1 homopentamer, β2 homopentamer, and GABA_A_ receptor heteropentamer were compared with the AlphaFold2 protein structures. The highest-ranked models were inputted into UCSF ChimeraX software (Ver. 1.8) to generate a molecular map (molmap) that could match the shapes of the EM images. Since the resolution of the α1 homopentamer is the highest, four EM images were chosen for comparison, while two images of the β2 homopentamer and the GABA_A_ receptor heteropentamer were used. The α1 homopentamer models show high similarities with the EM images, as seen in Figure 5a–d, with different points of view of the protein structure. The β2 homopentamer has a rough structure of a pentamer and the side view shows a similar shape; due to the low resolution of the EM images obtained, however, it was difficult to model and match them with the AlphaFold2 models. The GABA_A_ receptor heteropentamer only had top and bottom views of the pentamer and not enough images of the side view. Nevertheless, the two images chosen successfully show the pentameric structure and a central cavity in the protein structure, which is also modelled by AlphaFold2. Interestingly, the protruding residues of the γ2 subunit also seem to be shown on the EM images (red arrow in Figure 5g,h), suggesting that the γ2 subunit fragment obtained in the experiment can indeed bind and form heteropentamers. Overall, the resolution of the EM images is not exceptionally high, and the large number of particles used to determine the shape may be susceptible to noise. However, the EM images highlight the subunits’ ability to form oligomeric structures that resemble a channel protein with a clear central cavity.

### 2.6. Conserved Aromatic Residues Involved in Structural Motifs and Stabilisation of Subunits

**Figure 6:**
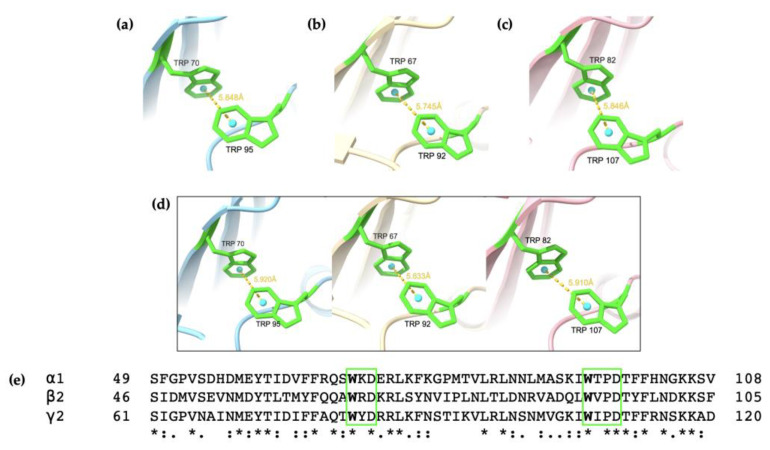
The conserved tryptophan (Trp, W) pair in the α1, β2, and γ2 subunits predicted by AlphaFold2. The conserved Trp residues in (**a**–**c**) homopentamers and (**d**) the heteropentamer subunits are predicted to be in proximity to each other, with distances of less than 6 Å apart, as depicted with ChimeraX. The α1, β2, and γ2 subunits are coloured in blue, yellow and pink respectively, with the Trp residues highlighted in green. The cyan circle is a pseudo-atom for measurement of the distance between the aromatic rings, which is depicted with a yellow dotted line. (**e**) Sequence comparison of the α1, β2, and γ2 subunits show the WxD and WxPD motifs, highlighted in green. The pair of conserved Trp residues are bolded. The conserved Trp residues are all 25 residues apart. The sequences are numbered without the signal peptide, Gln28 as Gln1 in the α1 subunit, Gln25 as Gln1 in the β2 subunit, and Gln40 as Gln1 in the γ2 subunit. Asterisks (*) show conserved and identical sequences, colon (:) show conservative substitution, and dot (.) shows semi-conservative mutations. Alignment by MAFFT in CLUSTAL format.

Since aromatic amino acids are frequently involved in both protein structural formation and ligand binding [24,25], the aromatic residues of the AlphaFold2 models were investigated. Tryptophan (Trp, W) is one of the least common amino acids, thus it is natural to investigate all the Trp residues in the subunit fragments. Observation identified two Trp residues in close proximity to each other in all three subunit fragments. Analysis showed that these two residues are the identified pair of Trp residues conserved in the extracellular domain (ECD) of Cys-loop receptors of the WxD and WxPD motifs [45]. As shown in Figure 6, the Trp pair in the predicted α1, β2, and γ2 homopentamers’ subunits have distances of 5.85 Å, 5.75 Å, and 5.85 Å, respectively; while the α1, β2, and γ2 subunits in the predicted heteropentamer have distances of 5.92 Å, 5.63 Å, and 5.91 Å, respectively. All the Trp pairs are less than 6 Å apart, and some may say they are within structural contact [69], perhaps for establishing π–π stacking of aromatic rings. It is evident that these two conserved residues play a role in the formation of GABA_A_ receptors and may function to stabilize the ECD [45].

To further confirm that these two tryptophan residues interact in experimentally acquired structures, eight cryo-EM structures from RCSB PDB were examined by the Trp of their WxD and WxPD motifs. The eight structures with PDB IDs 6X3X, 6X3Z, 8DD2, 8DD3, 8G4N, 8SGO, 8VQY, and 8VRN all depict the heteropentameric structure of the α1β2γ2 GABA_A_ receptor. Obtained from four different studies, the receptors in these cryo-EM models are bound with various ligands and thus may be presented as slightly different to the Alphafold2 predictions obtained in the current study. Nevertheless, all eight cryo-EM structures examined from PDB contain the WxD and WxPD motif stacked in a similar fashion, as shown in Supplementary Figure S3. From Table 2, it is evident that the distances between the Trp aromatic rings predicted by AlphaFold2 are within the range of the distances measured by ChimeraX in the cryo-EM structures. This further emphasizes the importance of these two motifs in the structural folding of the subunit fragments.

**Figure 7:**
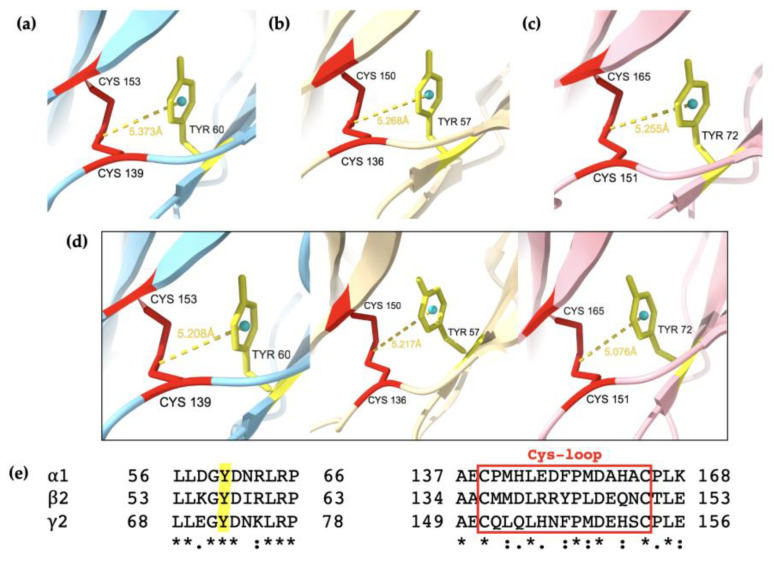
The conserved tyrosine (Tyr, Y) in the α1, β2, and γ2 subunits are packed on the Cys-loop. The conserved Tyr residues in (**a**–**c**) homopentamers and (**d**) the heteropentamer subunits are predicted to be in proximity to each other, with distances of less than 6 Å apart, as depicted with ChimeraX. The α1, β2, and γ2 subunits are coloured in blue, yellow and pink respectively, with the Tyr residues in yellow. The cyan circle is a pseudo-atom for measurement of the distance between the aromatic ring and the disulfide bond. (**e**) Alignment of the GYD sequence and Cys-loop sequence between the three subunits. Asterisks (*) show conserved and identical sequences, colon (:) show conservative substitution, and dot (.) shows semi-conservative mutations. Alignment by MAFFT in CLUSTAL format.

Previous studies suggest that Trp residues located in the Cys-loop’s proximity may affect the structural stability of the receptors [43,45]. In particular, a structural motif resembling an Ig-domain was described with a Trp residue packed against a conserved disulphide bridge, in this case, the Cys-loop [43,70]. As identified in Figure 1, the conserved Trp residue located at 18 residues on the C-terminal side of the second cysteine in the Cys-loop was examined in the AlphaFold2 models and the eight cryo-EM models. Although the Trp residue was positioned towards the inner fold of the subunits, it did not pack onto the Cys-loop as hypothesised (Supplementary Figure S4). Subsequently, the aromatic residues in the vicinity of the Cys-loop were further examined. It was found that a tyrosine (Tyr, Y) residue was packed on the Cys-loop, as seen in Figure 7. A corresponding S–π interaction was previously mentioned in a study on the structure of glycine receptors and suggests a structural role in conformational changes [71]. This interaction was also identified in the eight cryo-EM structures (Supplementary Figure S5), with similar distances observed (Table 3). Since the Tyr residues are part of a three-amino-acid conserved region between all three subunits, it may be hypothesized that this GYD sequence may be important to the structural assembly of the subunits. However, further experiments are needed to confirm the exact role of these residues. Perhaps the GYD sequence can be deleted to investigate the effects on protein folding as well as secondary and tertiary structures. This can also be performed together with ligand binding studies to explore the effects of the GYD sequence deletion on the conformational changes of the receptor.

### 2.7. Hydrophobicity Plot Indicates Subunit Fragments Are Relatively Hydrophilic

**Figure 8:**
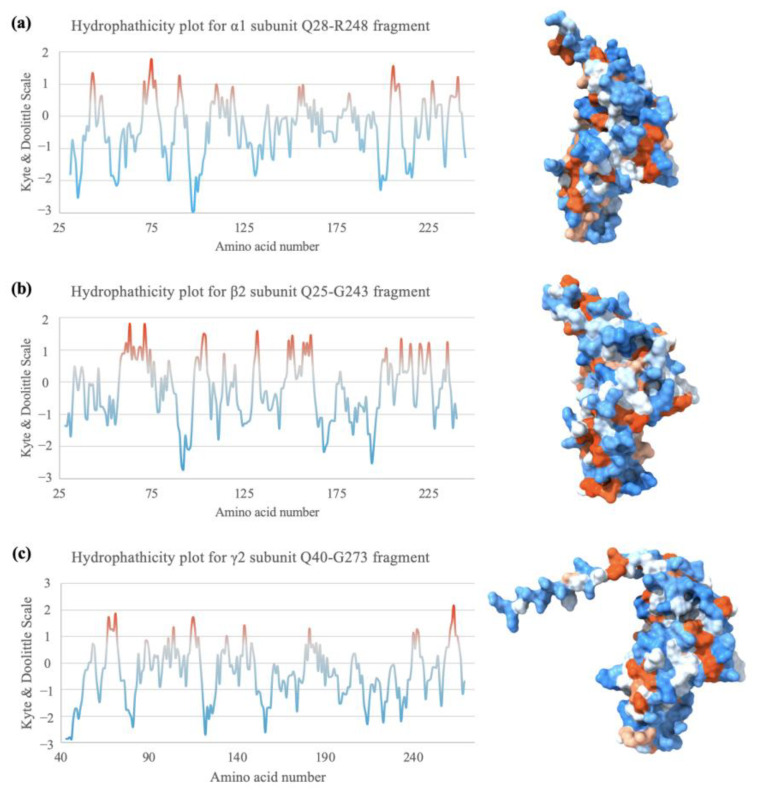
Hydrophobicity of the three subunit fragments. (**a**–**c**) Hydrophobicity plots according to the amino acid sequence of each subunit fragment. A negative score on the Kyte–Doolittle scale represents hydrophilicity and a positive score means the residue is relatively hydrophobic. Hydrophobicity surface depiction on the right, as portrayed by ChimeraX. Orange = hydrophobic residues, white = neutral residues, and blue = hydrophilic residues.

The GABA_A_ receptor subunits contain a hydrophilic extracellular domain (ECD) and four transmembrane domains. The fragments expressed in this study are all part of the ECD, thus it should be more hydrophilic on average [10,11]. Indeed, the hydrophobicity plots computed with ProtScale using Kyte–Doolittle analysis [72,73] in Figure 8 indicate that all three subunit fragments are relatively hydrophilic. In addition, according to the Kyte–Doolittle scale, a given segment with an average hydrophobicity score of +1.6 or above may suggest a possible transmembrane domain [74]. To confirm this on the AlphaFold2 model and test the accuracy of the models, the Gln28–Leu296 fragment of the α1 subunit [41,75] was computed with AlphaFold2 and ProtScale for an additional homopentamer prediction. AlphaFold2 prediction of the secondary structure is more than 97% identical with the shorter Gln28–Arg248 fragment, with one residue difference in the secondary structure at two positions, and a missing three-residue alpha helix structure (Supplementary Figure S6). A previous study indicated that the transmembrane domains TM1 and TM2 of the α1 subunit sequence correspond to the residues Phe254–Leu274 and Pro280–Arg301 [46]. The full TM1 segment and part of the TM2 are included in the additionally computed Gln28–Arg248 fragment. The hydrophobicity plot indeed had a much higher hydrophobicity in the transmembrane domain sequences but only with an average of around 0.95 for TM1 and 0.45 for part of TM2 (Supplementary Figure S7). However, only a partial segment of TM2 was computed, thus it may not be very reliable. Nevertheless, surface hydrophobicity may affect protein stability and the assembly of protein structures [76,77]. Hence, hydrophobicity studies may help determine the biochemistry of protein complex assembly.

## 3. Discussion

### 3.1. α1 and β2 Subunits of GABA_A_ Receptors Form Homopentamers

The current study successfully expressed and purified the Gln28–Arg248 fragment of the α1 subunit, the Gln25–Gly243 fragment of the β2 subunit, and the Gln40–Gly273 fragment of the γ2 subunit of the GABA_A_ receptor. Gel filtration chromatography, SDS-PAGE, and negative staining were carried out for each sample to confirm the sizes and purity of the proteins, as well as observe the aggregation levels. Results showed low protein aggregation and high homogeneity for the α1 subunit, but some level of aggregation was present for the β2 subunit, possibly due to issues related to the low protein abundance and strength of the detergent used, which will need further refinement. The abundance of the γ2 subunit seemed to be high from the SDS-PAGE results but was unable to form homopentamers in the current study. Yet it may also be due to possible detergent issues or complications during the protein refolding stage, hence more trials would be needed to purify both the β2 and γ2 proteins to confirm the results obtained in this study. Nevertheless, results from the negative staining and 2D classification of the α1 and β2 protein samples demonstrate that they can form rosette-like homo-oligomers, mostly homopentamers. A study has suggested the notion of α1 homopentamers formation as an intermediate structure before the assembly of α1β2γ2 GABA_A_ receptors [78]. A possibility can be considered that the formation of α1 and β2 subunit homo-oligomers may control the availability of subunits to the formation of the receptor. Understanding these mechanisms may also highlight key residues that may signal the assembly of the receptor [79]. This undoubtedly creates invaluable insights into the flexibility and complexity of GABA_A_ receptor formation to allow for the critical function of GABAergic transmission.

### 3.2. Evidence of Two β-Rich Domains Stabilised by Aromatic Residues in Each Subunit

**Figure 9:**
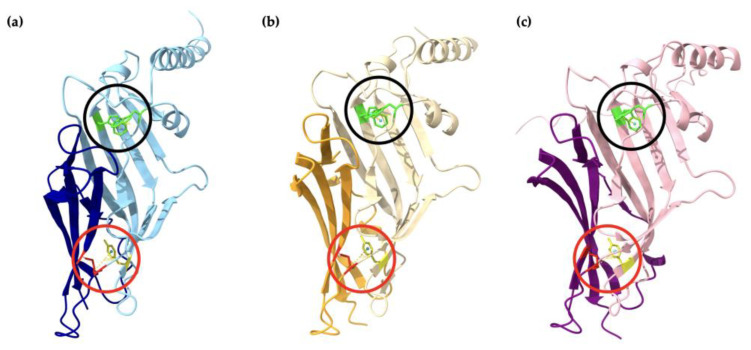
The α1, β2, and γ2 subunit fragments exhibit two β-rich domains stabilized by aromatic residue interactions. (**a**) The two domains of α1 subunit fragment Gln1–Glu138 and Cys139–Arg221 are coloured in light blue and dark blue, respectively. (**b**) The two domains of β2 subunit fragment Gln1–Ala135 and Cys136–Gly219 are coloured in yellow and orange, respectively. (**c**) The two domains of γ2 subunit fragment Gln1–Ala135 and Cys136–Gly219 are coloured in pink and purple, respectively. The disulfide bridge (red) with packed Tyr residue (yellow) is circled in red, while the two stacked Trp residues (green) are circled in black.

The AlphaFold2 predictions indicated that the overall structure of the extracellular domain (ECD) subunit fragments was β-rich. From Figure 4, the α1, β2, and γ2 subunit fragments exhibited 46.2%, 46.6%, and 44.4% β-sheet, respectively. It has previously been suggested that the α1 subunit ECD consists of two separate β-rich domains with different characteristics [43]. The separation of the two domains occurs at the first cysteine of the Cys-loop, with the C-terminal end domain as the membrane-proximal β-rich (MPB) domain [43]. Applying this to the AlphaFold2 predictions, the two domains on the α1 fragment can be identified as Gln1–Glu138 and Cys139–Arg221. From sequence alignment in Figure 1, the corresponding residues would be Gln1–Ala135 and Cys136–Gly219 in the β2 subunit fragment, as well as Gln1–Glu150 and Cys151–Gly234 in the γ2 subunit fragment. Figure 9 shows the two domains in each subunit and the aromatic residue interactions as observed in the current study. The Trp–Trp pair in the WxD and WxPD motifs can be seen as stabilising the first domain of the subunits. The Cys-loop and Tyr interaction may play a role in stabilising the Cys-loop structure or even function as an interconnection between the two domains to affect conformational changes. Perhaps it can be hypothesized that another aromatic residue is responsible for the stabilization of the structures within the MBP domain itself. The prediction from Alphafold2 can be strengthened with additional computational studies, such as molecular dynamics simulation to improve the thermal stability of the AlphaFold2 predicted structures [80]. This can allow the residues to arrange themselves into more stable positions and optimize the structure of the homopentamer. Overall, these findings from AlphaFold2 predictions can provide ideas to establish future hypotheses for testing.

### 3.3. β2 Subunit Fragments Homopentamers Possible Assembly Mechanism with Hydrogen-Bonds Identified

**Figure 10:**
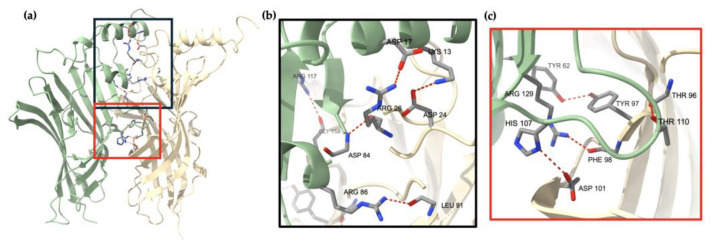
Inter-residue hydrogen bonds (H-bonds) identified in AlphaFold2 prediction of β2 subunit homopentamer. (**a**) β2 homopentamer with two subunit fragments shown (green and yellow), the other three chains are hidden for clarity. Black and red squares correspond to the (**b**,**c**) two detailed architectures of H-bonds between two chains of the β2 subunit homopentamer. Residues involved are shown with nitrogen and oxygen atoms labelled blue and red respectively. A total of nine H-bonds were identified. Each chain is linked to its neighbour by H-bonds depicted as red lines between residues. Residues are numbered based on β2 subunit Gln25 as Gln1, visualised in ChimeraX.

A previous study showed that the GABA_A_ receptor β3 subunit can form homopentamers [55]. Sequence alignment of the β2 and β3 subunits show that the two sequences are 89.95% identical (Supplementary Figure S8). Subsequently, theAlphaFold2 prediction of the β2 subunit homopentamer was compared with the β3 subunit crystal structure (PDB:4COF). As shown in Figure 10a,b, the six hydrogen bonds (H-bonds) formed between the residues Arg26 and Asp17, Tyr62 and Tyr97, Asp84 and Arg26, Arg86 and Leu91, Thr110 and Thr96, as well as Arg117 and Gly158, all correlate with Miller and Aricescu’s study. The three other pairs of H-bonds identified in the β2 subunit that were not present in the β3 subunit homopentamer were between Lys13 and Asp24, His107 and Asp101, as well as Arg129 and Phe98 (Figure 10a,c). The Lys13 and Asp24 are positioned near the N-terminus of the subunit fragment, which is also in proximity with the extra 10 amino acids in the γ2 fragment, as mentioned above. This further emphasises the potential importance of the 10-amino-acid insertion on the ability of the γ2 subunit to form homopentamers. Regardless, these three additional H-bonds may be worth investigating in future experiments to further explore the mechanisms of β2 subunit’s assembly into homopentamers.

### 3.4. AlphaFold2 Analysis Provide Insights into γ2 Subunit’s Inability to Form Homopentamers

**Figure 11:**
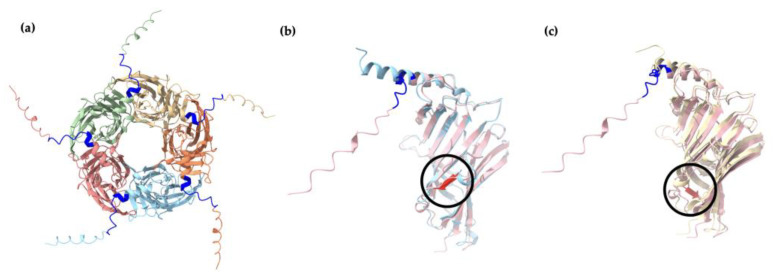
Two main structural differences of the γ2 fragment compared to the α1 and β2 fragments. (**a**) The γ2 homopentamer, as predicted by AlphaFold2 and visualised in ChimeraX. The 10 extra amino acid sequences that are not present in α1 and β2 subunits are highlighted in dark blue. Alignment of the (**b**) α1 and γ2 subunit fragments, as well as (**c**) β2 and γ2 fragments, were performed using Matchmaker in ChimeraX. The γ2 fragment is depicted in pink, while the α1 and β2 fragments are depicted in blue and yellow, respectively. The extra beta sheets in α1 and β2 subunits are circled and depicted in red. Root mean square deviation (RMSD) measuring similarity across all pairs in α1 and γ2 fragment alignment = 1.670 Å; RMSD across all pairs in β2 and γ2 fragment alignment = 2.528 Å.

The secondary structure prediction in Figure 4 indicates twelve beta-sheets in both the α1 and β2 homopentamers but only eleven beta-sheets in the γ2 homopentamer. Apart from this, there is also an ambiguity in the γ2 subunit structure computed by AlphaFold2 near the location of the extra 10-amino-acid sequence located at the N-terminal of the γ2 homopentamer. The two different secondary structures of the γ2 fragment may indicate that the possible conformational changes necessary for the assembly of homopentamers are not induced [78]. This aligns with the previous studies which showed that the expression of the γ2 subunit alone could not produce functional GABA receptors [81,82,83,84]. Figure 11 shows that the identified differences in the secondary structures are within the γ2 subunit’s polymerisation interface. Further investigations, both experimental and computational, are needed to uncover possible reasons why the γ2 subunits were unable to form homopentamers in the current study. For example, a deletion of the short 10-amino-acid sequence can be performed in vitro to validate the hypothesis. Furthermore, since the current study only uses fragments of the extracellular domain without the transmembrane domains, further studies can be performed by utilizing the full-length subunits to see if there are changes in structural characteristics.

### 3.5. Complexity of γ2 Subunit of the GABA_A_ Receptor

Since no two of the same subunits are adjacent to each other in the predominant GABA_A_ receptors, it has been suggested that a homo-oligomeric formation may be a preferred intermediary mechanism when other types of subunits are absent [78]. If it is eventually confirmed that γ2 does not form homo-oligomers, perhaps it can be further hypothesized that γ2 is the last subunit to be incorporated into the receptor as there are no necessary intermediary structures. Although the ability of γ2 subunit to form homopentamers is yet to be determined, their function in the GABA_A_ receptor is undeniably crucial.

Multiple studies have elaborated on the relationship between γ2 and receptor trafficking [85], especially in its relationship with gephyrin [86,87,88,89,90]. In particular, one study indicated that the fourth transmembrane domain of the γ2 subunit is essential for the postsynaptic clustering of GABA_A_ receptors [89]. Some studies indicate a role of the α2 subunit in binding gephyrin [91,92], but only the γ2 subunit was shown to rescue postsynaptic clustering of GABA_A_ receptors in γ2^−/−^ neurons [89]. It can be further hypothesized that the γ2 subunit is the limiting factor of postsynaptic clustering of GABA_A_ receptors thus supporting the initial hypothesis of the γ2 subunit being the last subunit to be assembled as, once the γ2 subunit is present, postsynaptic clustering of the predominant receptor will occur. Regardless, many more studies are needed to investigate these hypotheses and thoroughly explore the assembly mechanisms of GABA_A_ receptors. Understanding these mechanisms may also provide invaluable insights into the trafficking mechanism of similar receptors, as well as their ligand binding abilities.

### 3.6. Constructed α1β2γ2 Heteropentameric GABA_A_ Receptors

Apart from homopentamer construction, the expressed fragments of α1, β2, and γ2 subunits were combined in the predominant 2:2:1 ratio to study the oligomeric structure. Results indicated that subunits combined in this ratio were able to form heteropentamers in a stable manner (Figure 3g). This confirms that the three subunit fragments expressed in the study can form heteromeric structures with each other. Although the 2:2:1 pentamers had stable protein structure, negative staining results showed some level of aggregation, and the sample concentration was relatively low. Therefore, further investigations are needed to obtain samples with higher concentration and purity. Overall, the protein samples still provide a useful model for studying the homopentameric nature of the receptors. In our future studies, a higher resolution of the GABA_A_ receptors’ structure will be needed to obtain the atomic structure of the receptors and identify the key residues of drug binding.

Heteropentameric GlyR has been suggested to be formed in 2:3 or 3:2 ratios of α and β subunits, respectively [90,93], thus this study also aimed to find out whether the GABA_A_ receptors α and β subunits can form heteropentamers in similar 2:3 and 3:2 ratios. However, no protein complexes were seen in combining the two subunits. Since these combinations may not be naturally present in the human body, perhaps AlphaFold2 can first be used to model the structures and predict the possibility of them forming pentamers. This not only aids in the understanding of subunit binding mechanisms of the GABA_A_ receptor but may also indicate the essential role of γ2 subunits in receptor formation—if α and β subunits truly do not prefer to form pentamers without a third subunit type.

### 3.7. Potential of Using AlphaFold for Aided Experimental Design and Evaluation

Since the launch of the AlphaFold2 system and the release of the open-source software, its potential for various applications in biology and medicine has allowed it to gain a reputation. It has been suggested that AlphaFold2 can be harnessed in processes such as drug discovery [94] and modelling protein–protein interaction [95,96]. Still, the majority of studies apply AlphaFold2 as an addition to experimental studies to aid the determination of protein structures. This prediction algorithm can be used for homology modelling, de-novo modelling, and ML-based modelling [97]; most importantly, the predicted structures can be used along with other structural protein techniques to accelerate the process of protein structure identification. There have been numerous successful studies utilizing AlphaFold2 with X-ray crystallography [98,99] and cryo-EM [100,101,102,103]. Although the system has been proven to be a useful tool in the structural biology field, it is still crucial to perform experiments to confirm the predictions. Accordingly, the conclusive structure of the GABA_A_ receptor and its subunit homopentamers can only be confirmed by more structural studies such as X-ray crystallography, cryo-EM, or mass spectrometry [53], especially when studies are needed to mimic the native environment of the receptor. Ultimately, studies can be performed to analyse ligand and drug binding sites on the GABA_A_ receptor structures predicted by AlphaFold2 or even AlphaFold3. The new AlphaFold3 has been shown to have improved accuracy on structural predictions and ligand docking simulations [104]. Combined with experimental studies, AlphaFold3 have the potential to elucidate the mechanisms of GABA_A_ receptor formation and the comprehensive structural changes during ligand binding processes.

An increasing number of studies have revealed the association of GABA_A_ receptors with various neuropsychiatric disorders, such as schizophrenia, epilepsy, and Alzheimer’s disease [105,106,107,108]. Thus, understanding the mechanisms of the GABA_A_ receptor is crucial for combatting these central nervous system (CNS) disorders. Ultimately, a high-resolution structure for an important family of neurotransmitter receptors at the atomic level will pave the way to new drug design and therapeutics for various neuropsychiatric diseases.

## 4. Materials and Methods

### 4.1. Sequence Alignment

The protein sequences of GBRA1 (P14867), GBRB2 (P47870), and GBRG2 (P18507) were obtained from UniProt [66]. Sequence alignment and percent identity matrix of the α1, β2, and γ2 subunit protein fragments were generated by CLUSTALW format alignment by MAFFT FFT-NS-i (Ver. 7.487) on the EMBL-EBI website [109,110].

### 4.2. Buffers

Methods are adapted from Xue et al. [75].

The truncated protein samples of each subunit were obtained as inclusion bodies and refolded into their active forms using buffers. Then, the proteins were further purified in soluble forms using different detergents, aiding the proper refolding and improving the homogeneity of the samples for further high-resolution studies. The buffers used were as follows:

- Wash Buffer A: 50 mM Tris-Cl, pH 8.0 and 10 mM EDTA.
- Lysis buffer: 100 mM Tris-Cl, pH 8.0, 10 mM EDTA 5 mM DTT, 100 mM NaCl, 10% Glycerol and 200 µg/mL lysozyme.
- Wash Buffer B: 100 mM Tris-Cl, pH 8.0, 10 mM EDTA 5 mM DTT, 100 mM NaCl, 10% Glycerol 2M urea and 2% deoxycholic acid.
- Elution Buffer: 10 mM glycine pH 10.3 and 2% sodium dodecyl sulfate (SDS).

### 4.3. Cloning and Protein Expression

A prokaryotic expression vector, specifically the pTrcHis vector, was used to introduce the bovine gene of interest into *E. coli* strain—NovaBlue—to express the proteins. The culture was prepared using 1 mL stock of the competent cells, 100 mL of LB broth, and 100 μg/mL of ampicillin. The culture was incubated overnight at 37 °C on a shaker and then transferred into 1 L baffled bottles containing LB broth and 100 μg/mL ampicillin. Optical density (OD) at 600 nm was observed until it reached between 0.4 and 0.5, where it was then induced with IPTG with a final concentration of 0.8 mM. Cells were collected after 3–4 h of growth in a 37 °C shaker. Cells were harvested by centrifugation at 1300× *g*, 4 °C for 20 min. The pellet containing the bacterial cells was washed with 200 mL of wash buffer A and centrifuged again at 2250× *g*, 4 °C for 20 min. The cell pellet was kept at −20 °C before purification.

### 4.4. Cell Purification

The bacterial cell pellet was resuspended in 100 mL lysis buffer and shaken for 1 h at 37 °C. Cells were centrifuged at 10,500× *g* at 4 °C for 20 min, and the supernatant was discarded. The pellet was resuspended in 150–180 mL wash buffer B and sonicated for 30 s then centrifuged at 10,500× *g* at 4 °C for 20 min, and the supernatant was discarded. The cell lysate was washed with the same experimental steps with wash buffer B for 4 times. The pellet was washed with 200 mL freshly prepared 4 M urea, followed by 200 mL Milli-Q water, and centrifuged at 10,500× *g* at 4 °C for 20 min after each wash. Then, 20 mL of freshly prepared 7 M guanidine hydrochloride (GnCl) was added to the lysate and shaken at room temperature for 1 h. Centrifuge again at 20,000× *g* and 4 °C for 20 min. The function of GnCl was to solubilise the inclusion bodies and allow the protein to bind with water. After washing with GnCl, the supernatant was kept instead of the pellet. To form precipitation of the protein, 5.3 g of ammonium sulphate (NH_4_)_2_SO_4_ (final concentration 2M) was gradually added into the supernatant with stirring to ensure that it dissolved. The solution was left at room temperature for a minimum of 4 h or even overnight, then the precipitate was washed with ice-cold distilled water 3 times and centrifuged at 20,000× *g*, 4 °C for 15 min. The protein pellet was collected and solubilized in 4% SDS and 100 μL β-mercaptoethanol, followed by shaking overnight.

### 4.5. Protein Refolding

After solubilization, a 0.22 µm syringe filter was used to remove large particles from the pellet. The filtered pellet was loaded into a Superdex 200 HR 26/60 column (Amersham Pharmacia Biotech, Uppsala, Sweden) for gel filtration chromatography. The samples were collected by running elution buffer into the column at a rate of 1 mL/min with a Bio-Rad EP-1 Econo Pump (Bio-Rad, Hercules, CA, USA). Eluents were collected in numbered tubes in a Bio-Rad Model 2110 Fraction Collector (Bio-Rad, Hercules, CA, USA). A NanoDrop Spectrophotometer (Thermo Fisher Scientific Inc., Waltham, MA, USA) was used to measure the concentration of proteins at A_280_ in each tube. The samples with higher protein concentrations were selected and used for 12% SDS-PAGE with Coomassie brilliant blue R-250 stain. After SDS-PAGE, samples that were confirmed to have the correct size and higher purity were dialysed for the negative staining.

### 4.6. Constructing Heteropentamers

Other than the individual subunits, the samples were also combined into a GABA_A_ receptor pentamer with a 2:2:1 ratio before column elution to investigate their ability to form heteropentamers. First, 2 mL of the α1 subunit, 2 mL of the β2 subunit, and 1 mL of the γ2 subunit of the same were combined and put into the shaker for one hour to allow them to interact and form oligomers. Then, 10% SDS-PAGE was performed on the complex.

Previously, studies showed that α1 and β3 subunits formed pentamers in vitro [111] and the α1 and β2 subunits combinations produced functional surface expression [34,112,113]; hence, in this study, the α1 and β2 subunit fragments were also combined in 2:3 and 3:2 ratios to attempt pentameric formation. Unfortunately, the pentameric formation was not achieved and no structures were seen.

### 4.7. Proteins Dialysis

Protein samples were transferred into semipermeable membranes submerged in different concentrations of sodium dodecyl-sulfate (SDS) and Glycine solutions. Firstly, the samples were submerged in 2 litres of 1.5% SDS with 10 mM Glycine (pH 10.3) then transferred to 2 litres of 1.0% SDS with 10 mM Glycine. Then, gradual dilutions of 0.8% SDS → 0.6% SDS → 0.4% SDS → 0.2% SDS were prepared, each with 10 mM Glycine. Lastly, dialysis was conducted in a 4 × 4 litre pure 10 mM Glycine solution (pH 10.3) for 4 days. The protein samples were then filtered, and the optical density and molecular weight were checked before negative staining.

### 4.8. Negative Staining of the Protein

The formvar, carbon-coated copper grid (300 meshes, manufactured by Ted Pella, Redding, CA, USA) used for negative staining was hydrophilized using a plasma cleaner and vacuumed for 2 min. After that, both hydrophilization and vacuum pump were turned off until the balance was completed, and the copper grid was removed. Next, 2.5 µL of the protein sample was loaded into the formvar, and the carbon-coated copper grid used for negative staining was hydrophilized using a plasma cleaner and vacuumed for 2 min then incubated for 1 min. Then, 4 µL of 2% uranyl acetate was added and incubated for 1 min. Excess liquid was absorbed using filter paper. Only a thin layer of uranium acetate was retained, dried, and examined by Talos L120C Transmission Electron Microscope (TEM) manufactured by Thermo Fisher Scientific Inc. (Waltham, MA, USA), operated at 120 kV. Particles were manually picked for 2D classification in Relion (Ver. 3.1.1) [114]. To generate 2D averages, around 900–950 particles were manually selected from 20 micrographs for each structure. No 3D reconstruction was performed in this study. 

### 4.9. Computational Prediction of Protein Structure Using AlphaFold2

The sequence of the fragments was obtained from UniProt [66] with IDs P14867, P47870, and P18507 for α1, β2, and γ2 subunits, respectively. The sequences for each subunit fragment were applied to the open-source AlphaFold2 interactive Python Notebook (Python Ver. 3.10.12) in the Google Colab [57]. Sampling options were edited based on a few parameters. Recycling used the previous output for the next iteration’s input. The number of recycling would stop after a certain tolerance level (tol) had been reached, which was set to a root mean square deviation (RMSD) of 0.5 Å. When subsequent predictions would reach the threshold of 0.5 Å difference from the previous prediction, recycling would terminate, and a predicted model would be generated. The predicted local distance difference test (pLDDT) score shows the confidence level of the prediction. The estimate of template modelling score (pTM) assesses the topological similarity of protein structures. It is suggested that the multimer interface pTM score (ipTM) score can have a confidence cut-off of 0.75 [115]. AlphaFold2 Multimer_v3 Model was used at the time of performing this study. The highest-ranked prediction models were then analysed in UCSF Chimera software (Ver. 1.17.3) for the pipes and plank models and UCSF ChimeraX (Ver. 1.8) for other structural analyses [67,68,116]. The matchmaker function in ChimeraX was utilised to align the homopentameric structures. Chain pairing was performed using the best-aligning pair of chains between the reference and match structure. Alignment and fitting were at default settings, with the iteration cutoff distance set as 2.000 Å.

### 4.10. Comparison of Negative Staining Electron Microscopy (EM) Images and AlphaFold2 Predicted Structures

The predicted models’ structures were compared with negative staining EM images. The resolution of negative staining EM is about 10–20 Å [117], thus density maps of 10 Å and 15 Å were generated using molmap command in ChimeraX. The best-fit resolution was used for comparison. The EM micrographs of α1 homopentamer and α1β2γ2 heteropentamer had a relatively higher resolution than the β2 homopentamer. Hence, 10 Å molmaps were used for the α1 homopentamer and α1β2γ2 heteropentamer, while 15 Å molmaps were used for the β2 homopentamer comparison.

### 4.11. Comparison of Existing Cryo-EM Models and AlphaFold2 Predicted Structures

Eight cryo-EM structures from RCSB PDB were compared to the AlphaFold predicted structures. The eight structures with PDB IDs, 6X3X, 6X3Z, 8DD2, 8DD3, 8G4N, 8SGO, 8VQY, and 8VRN, all depict the heteropentameric structure of the α1β2γ2 GABA_A_ receptor. The structures were selected from four different studies and with different ligands to increase variety. The structures were extracted in ChimeraX with PDB ID and examined for the WxD and WxPD Trp pair, Cys-loop and Tyr stack, as well as H-bonds between subunits. A pseudo-atom was created in the centre of the aromatic rings of the Trp and Tyr residues to measure the distances of potential π-stacking.

### 4.12. Hydrophobicity Plots

Hydrophobicity plots were generated with Protein Identification and Analysis Tools on the ExPASy Server—ProtScale [73]. The UniProt/SwissProt accession numbers P14867, P47870, and P18507 were used to retrieve the amino acid sequences of α1, β2, and γ2 subunits, respectively, and the amino acid range was selected according to each fragment. The window size of 7 was used for the subunit fragments, as it was aimed at finding hydrophilic regions exposed on the surface and may potentially be antigenic [73]. In this case, the extracellular domain regions exposed on the cell surface were related to ligand binding. A higher window size of 19 was recommended to identify more hydrophobic residues and transmembrane domains [73]. Since the Gln28–Leu296 fragment included two transmembrane domains of the α1 subunit, window size of 19 was used for the additional hydrophobicity plot (Supplementary Figure S7).

## 5. Conclusions

The α1 and β2 subunit fragments expressed in this study successfully formed homo-oligomers, mostly homopentamers, as seen from negative staining EM results. However, it is still undetermined whether the γ2 subunit can form homo-oligomers like its counterparts. Efforts were also made to form the α1β2γ2 GABA_A_ receptors, and results show a pentameric structure with a central cavity. AlphaFold2 was used to model each pentameric structure to predict the structural characteristics and assembly mechanism of the subunits and the receptor. Results were compared with the previous cryo-EM studies to gain more insights into the possible assembly mechanism of an important neurotransmitter receptor that may affect ligand binding processes and drug development for neuropsychiatric diseases associated with the GABA_A_ receptor. The role of aromatic residues in the structural formation of the subunits was further clarified, suggesting functions in stabilising the two β-rich domains identified. AlphaFold2 predictions provide insights into γ2’s inability to form homopentamers. However, protocols will need to be optimised for protein purification to obtain a higher concentration for obtaining a high-resolution structure of GABA_A_ receptors using experimental studies. Further experiments can also be performed on the γ2 subunit to confirm its inability to form homopentamers. Computational studies such as molecular dynamics simulation can also be performed to strengthen the AlphaFold predictions. Overall, this study provides new insights into the formations and structural characteristics of GABA_A_ receptors.

## Figures and Tables

**Table 1:** Percent identity matrix for sequence alignment of GABA_A_ receptor α1, β2, and γ2 subunit protein fragments individually, as generated by MAFFT. Sequence homology is highest between α1 and γ2 fragments, lowest between α1 and β2.

	α1	β2	γ2
α1	100%	36.57%	51.83%
β2	36.57%	100%	39.45%
γ2	51.83%	39.45%	100%

**Table 2:** Distances (Å) between the two tryptophan (Trp, W) residues in the WxD and WxPD motifs in each subunit of the eight PDB cryo-EM structures examined.

PDB ID	α1 Subunit (Trp70 and Trp95)	β2 Subunit (Trp67 and Trp92)	γ2 Subunit (Trp82 and Trp107)
6X3X	6.01 Å	5.84 Å	5.89 Å
6X3Z	5.91 Å	5.83 Å	5.87 Å
8DD2	5.96 Å	5.71 Å	5.88 Å
8DD3	5.73 Å	5.68 Å	5.97 Å
8G4N	6.00 Å	5.90 Å	5.99 Å
8SGO	5.95 Å	5.80 Å	5.90 Å
8VQY	6.02 Å	5.71 Å	5.89 Å
8VRN	5.99 Å	5.66 Å	5.95 Å
Max	6.02 Å	5.90 Å	5.99 Å
Min	5.73 Å	5.66 Å	5.87 Å
Average (± SEM)	5.95 Å (±0.0343)	5.77 Å (±0.0308)	5.92 Å (±0.0159)

**Table 3:** Distances (Å) between the Cys-loop disulfide bond and the packed tyrosine (Tyr) residue in each subunit of the cryo-EM structures examined.

PDB ID	α1 Subunit (Cys-Loop and Tyr60)	β2 Subunit (Cys-Loop and Tyr57)	γ2 Subunit (Cys-Loop and Tyr72)
6X3X	4.11 Å	4.48 Å	5.13 Å
6X3Z	5.16 Å	4.56 Å	4.52 Å
8DD2	5.30 Å	5.02 Å	4.47 Å
8DD3	5.27 Å	5.25 Å	4.56 Å
8G4N	5.12 Å	5.32 Å	5.03 Å
8SGO	5.05 Å	4.68 Å	4.26 Å
8VQY	5.14 Å	5.34 Å	4.31 Å
8VRN	5.16 Å	5.29 Å	4.17 Å
Max	5.30 Å	5.34 Å	5.13 Å
Min	4.11 Å	4.48 Å	4.17 Å
Average (± SEM)	5.04 Å (±0.136)	4.99 Å (±0.129)	4.56 Å (±0.124)

## Data Availability

Data are contained within the article and Supplementary Materials.

## Supplementary Materials

The following supporting information can be downloaded at: https://www.mdpi.com/article/10.3390/ijms251810142/s1.
