# Supplementary material for: Modular Structure and Polymerization Status of GABAA Receptors Illustrated with EM Analysis and AlphaFold2 Prediction
Source: Int J Mol Sci. 2024 Sep 21;25(18):10142. doi: 10.3390/ijms251810142 (PMC11432007; doi:10.3390/ijms251810142)
Supplement: Supplementary file 1 [file ijms-25-10142-s001.zip › Supplementary File.pdf]

Supplementary File.

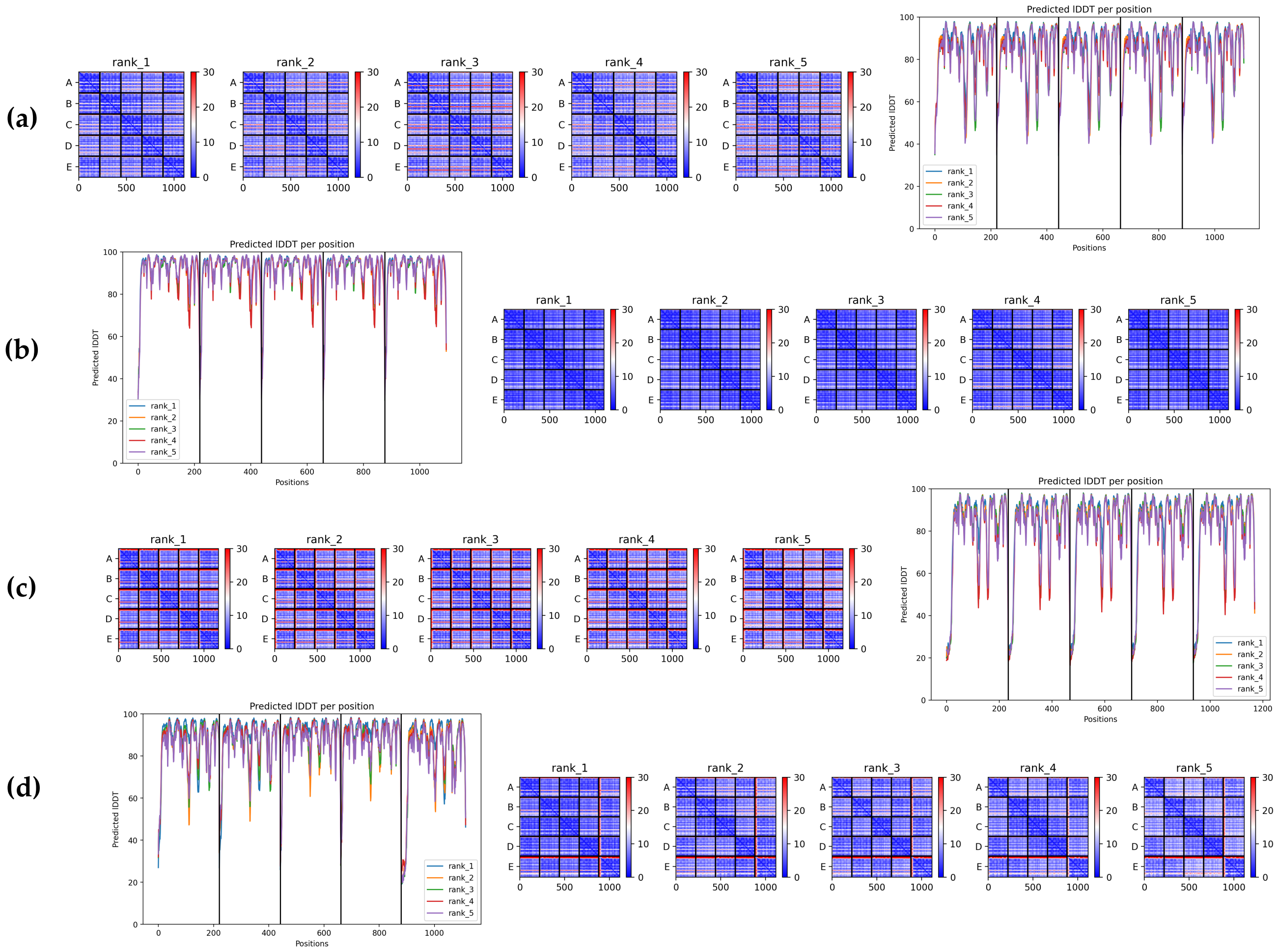

Supplementary Figure S1. Prediction quality and error estimates by AlphaFold2. Predicted Aligned Error (PAE) plots and predicted local distance difference test (pLDDT) per position. PAE heatmap shown with residue numbers along both axes, colours indicating PAE value for the pair of residues. Colour is scaled from 0 - 30Å, where low PAE of less than 5Å (blue) is optimal (higher confidence). Models with "rank_1" is the best predicted model and were used for further analysis with Chimera. Plots are shown for **(a)** α1 subunit Gln28-Arg248 fragment homopentamer, **(b)** β2 subunit Gln25-Gly243 fragment homopentamer, **(c)** γ2 subunit Gln40-Gly273 fragment homopentamer, and **(d)** native GABA(A) receptor with α1β2γ2 in a 2:2:1 ratio. pLDDT is lower for the first ~10-20 residues for all the predicted structures.

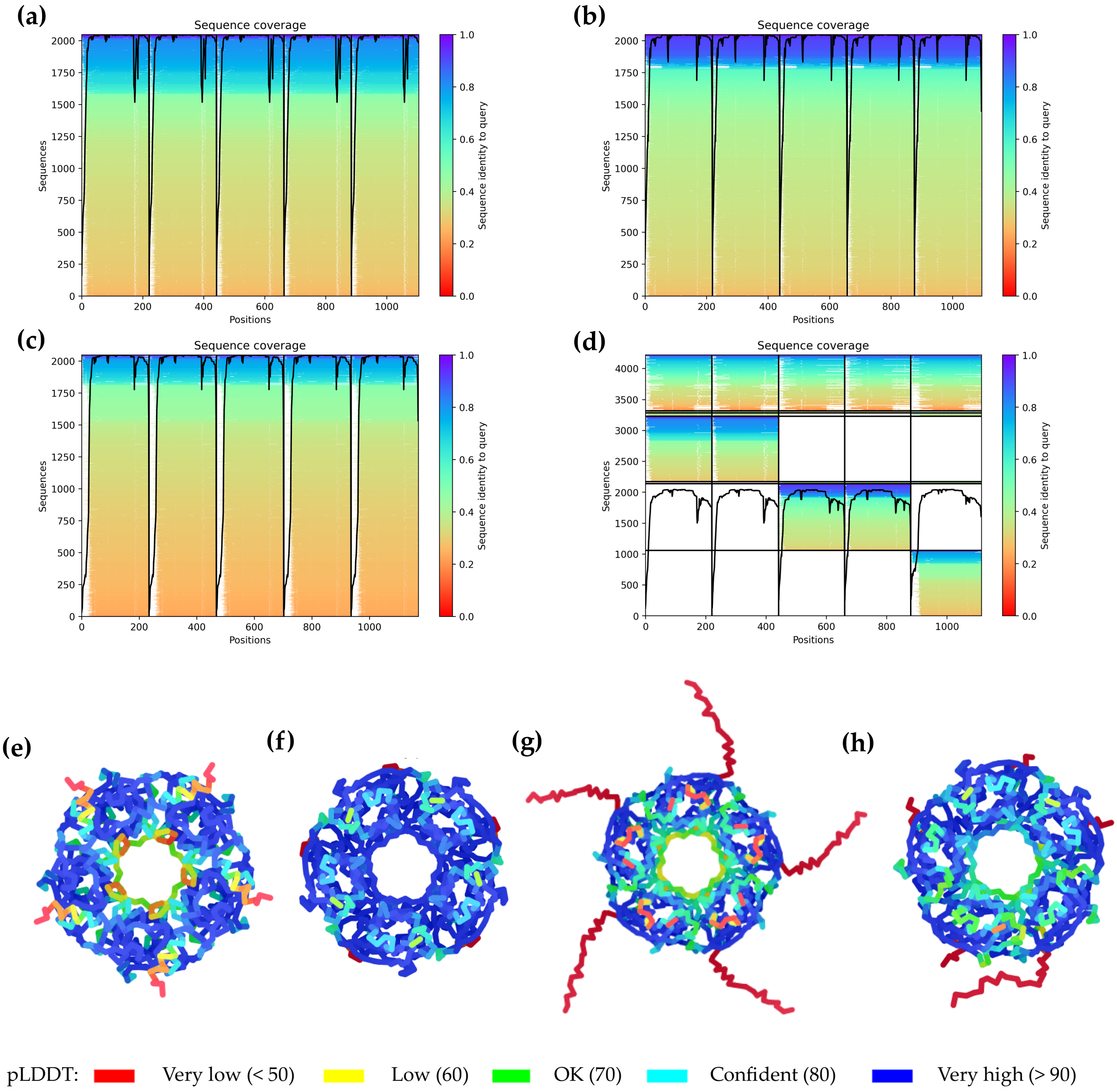

Supplementary Figure S2. AlphaFold2 multiple sequence alignment (MSA) and predicted pentameric structures coloured by pLDDT. MSA plots show sequence coverage for **(a)** α1 subunit Gln28-Arg248 fragment homopentamer, **(b)** β2 subunit Gln25-Gly243 fragment homopentamer, **(c)** γ2 subunit Gln40-Gly273 fragment, **(d)** homopentamer, and GABA(A) receptor. **(e)** α1 subunit homopentamer structural prediction, recycle=3, pLDDT=86.3, pTM=0.864, ipTM=0.845, tol=0.339. **(f)** β2 subunit homopentamer structural prediction, recycle=4, pLDDT=91.2, pTM=0.9, ipTM=0.885, tol=0.203. **(g)** γ2 subunit homopentamer, recycle=3 pLDDT=82.5 pTM=0.833 ipTM=0.818 tol=0.388. **(h)** GABA(A) receptor with α1β2γ2 in a 2:2:1, pLDDT=87.8 pTM=0.889 ipTM=0.882.

(a) 6X3X

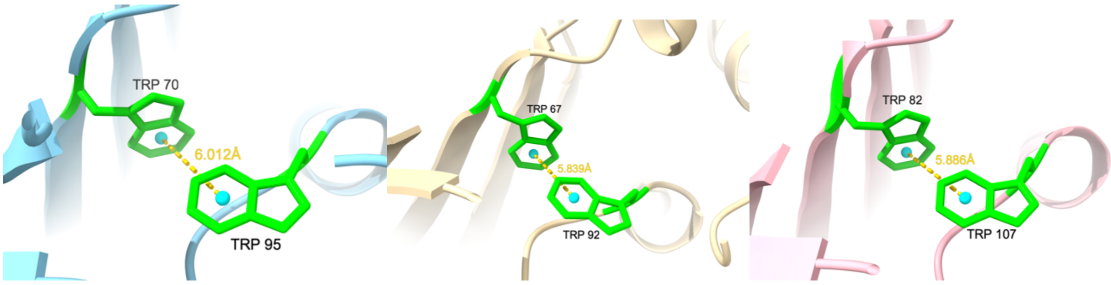

(b) 6X3Z

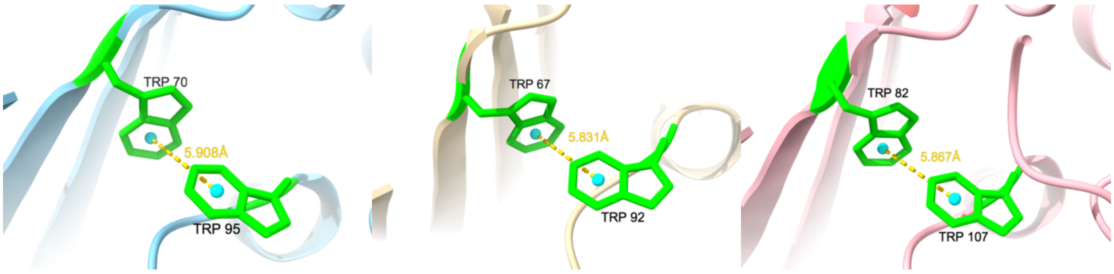

(c) 8DD2

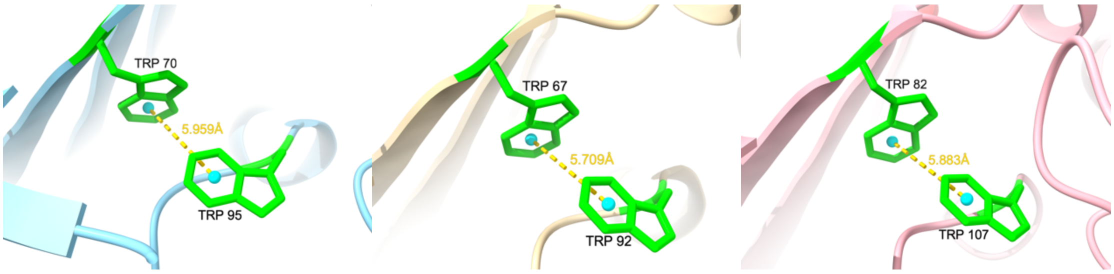

(d) 8DD3

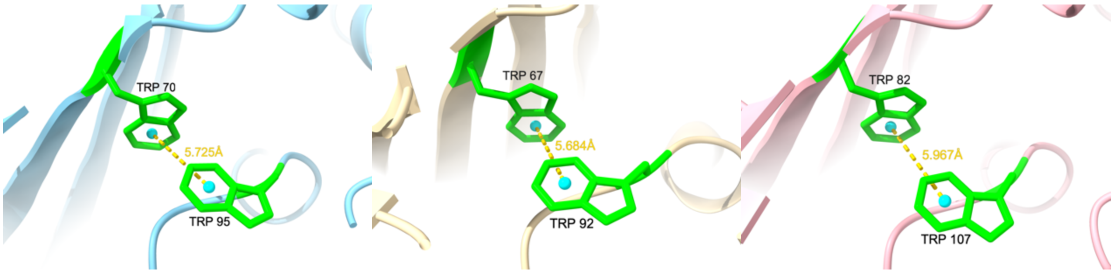

(f) 8G4N

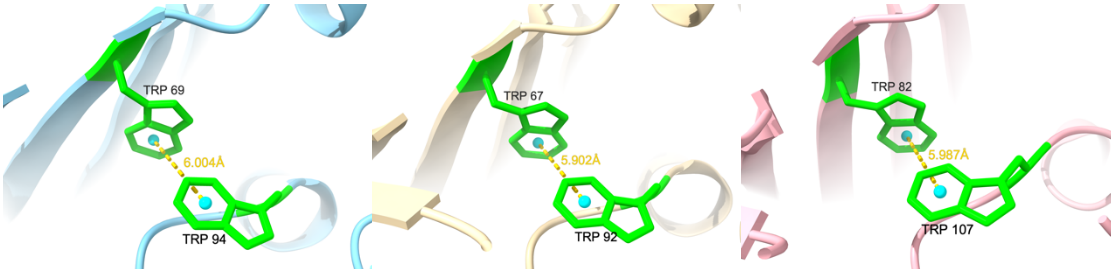

(g) 8SGO

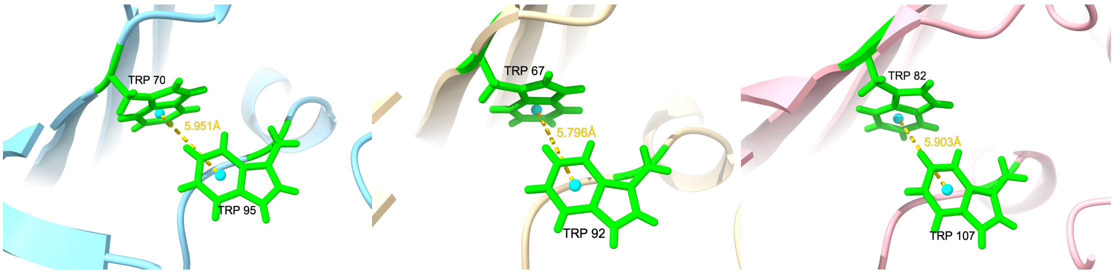

(h) 8VQY

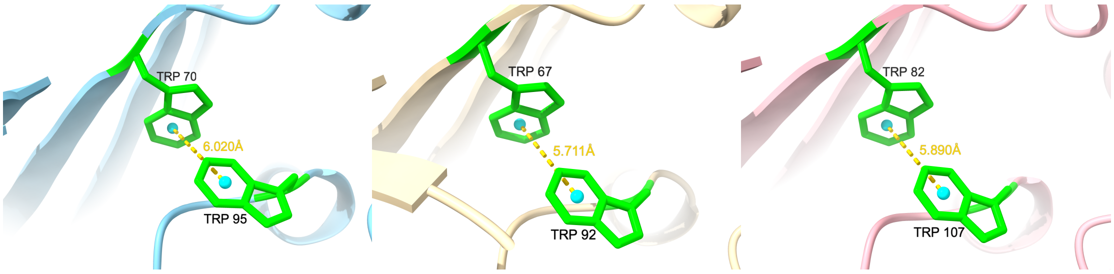

(i) 8VRN

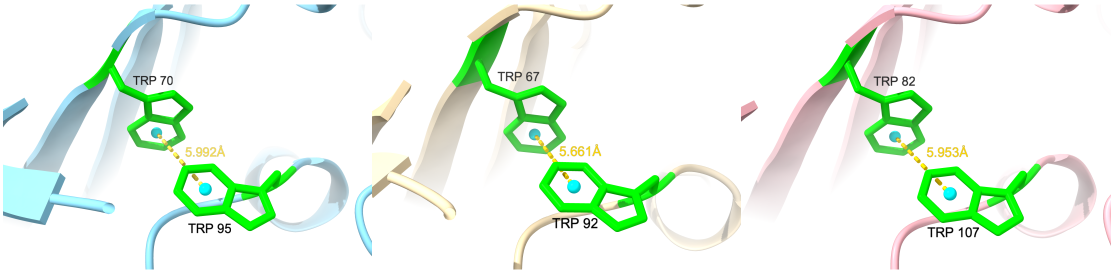

Supplementary Figure S3. Atomic distances between tryptophan residues on WxD and WxPD motifs of eight RCSB PDB cryo-EM structures. Structures with the PDB IDs 6X3X, 6X3Z, 8DD2, 8DD3, 8G4N, 8SGO, 8VQY, and 8VRN **(a-i)** were examined with ChimeraX (Ver. 1.8) for the tryptophan stacking between WxD and WxPD motifs. The α1 subunit, β2 subunit, and γ2 subunit are shown in sky blue, yellow, and light pink, respectively. The tryptophan residues are labelled and depicted in green with a pseudo-atom in the middle of the aromatic ring for measurement of the distance. The distances are shown in yellow with a dotted line between the pseudo-atoms.

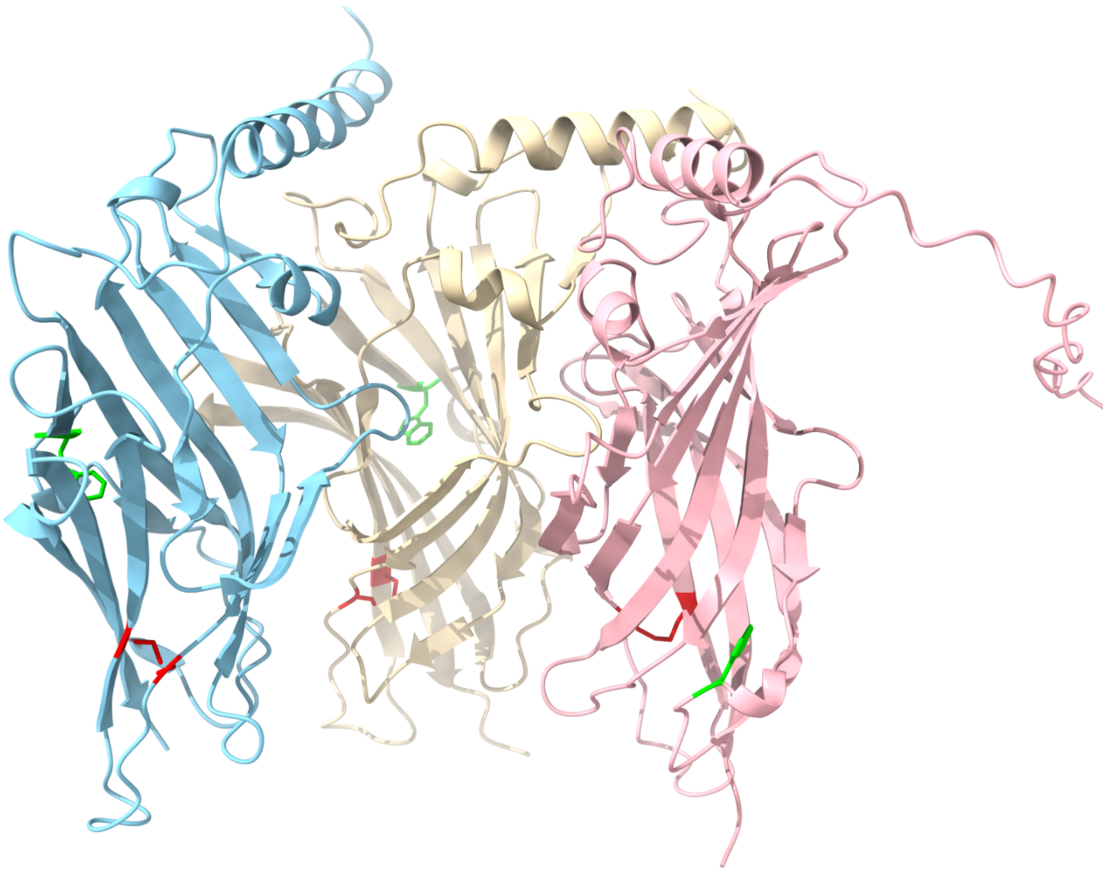

Supplementary Figure S4. The conserved Trp residue located at 18 residues on the C-terminal side of the second cysteine in the Cys-loop does not pack into the Cys-loop. The AlphaFold2 prediction of the α1β2γ2 GABAA receptor heteropentamer structure is shown, with the α1 fragment in blue, the β2 fragment in yellow, and the γ2 fragment in pink. The Cys-loop is colored red and the Trp residue is colored in green.

(a) 6X3X

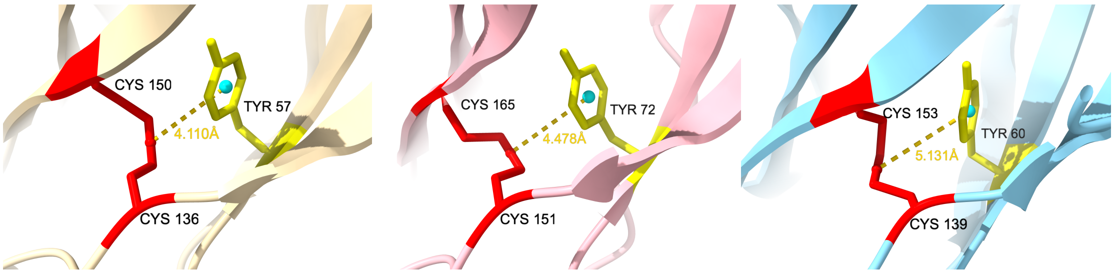

(b) 6X3Z

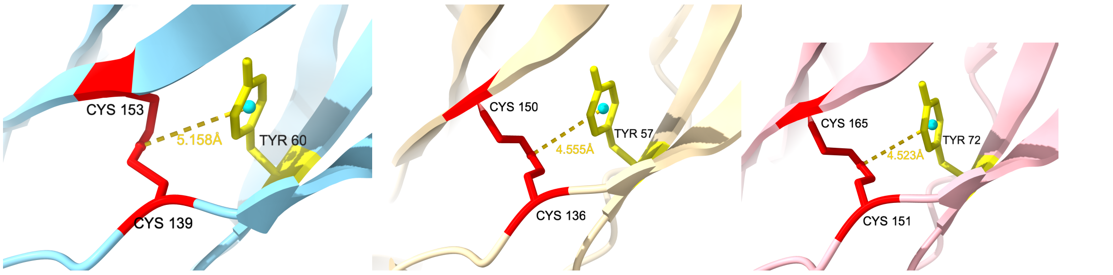

(c) 8DD2

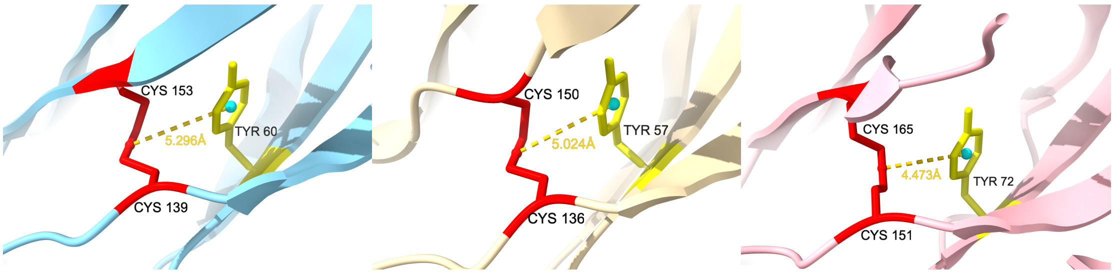

(d) 8DD3

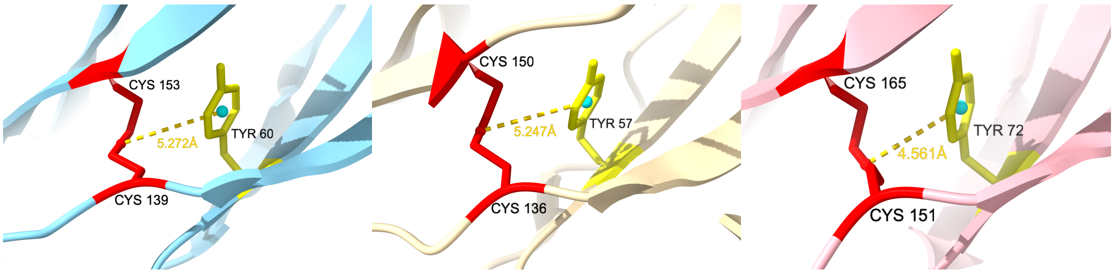

(f) 8G4N

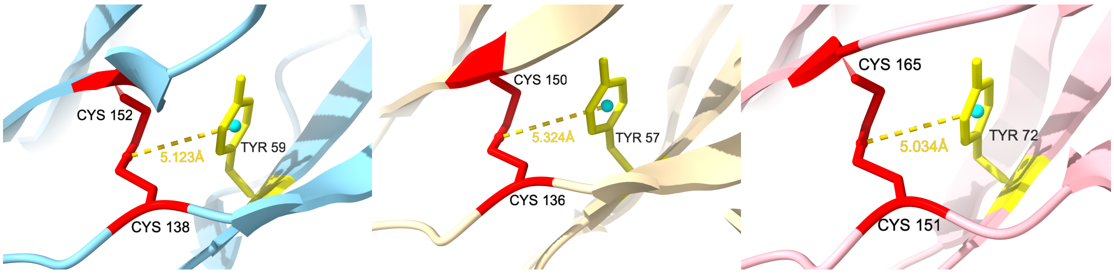

(g) 8SGO

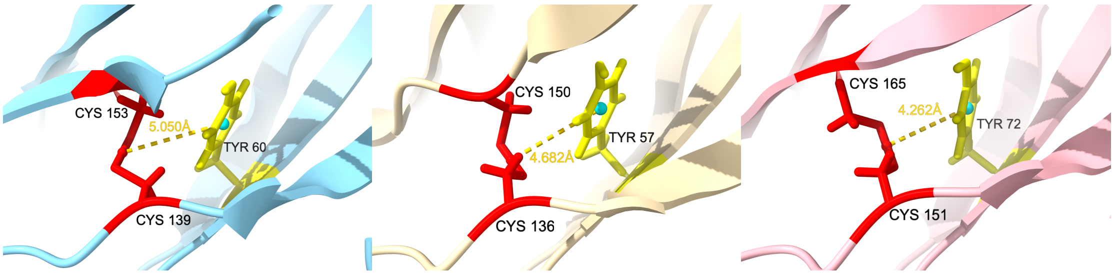

(h) 8VQY

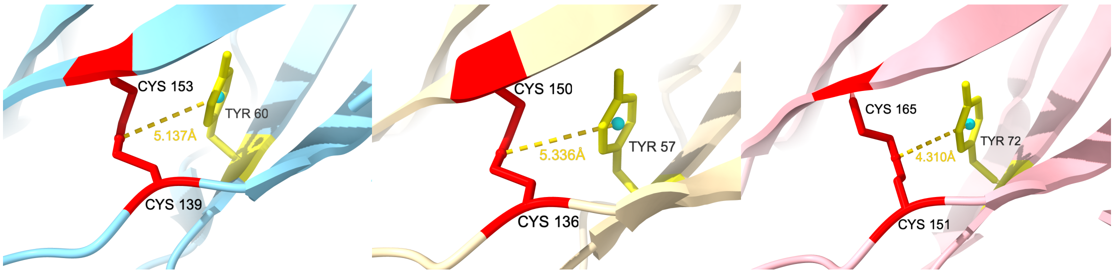

(i) 8VRN

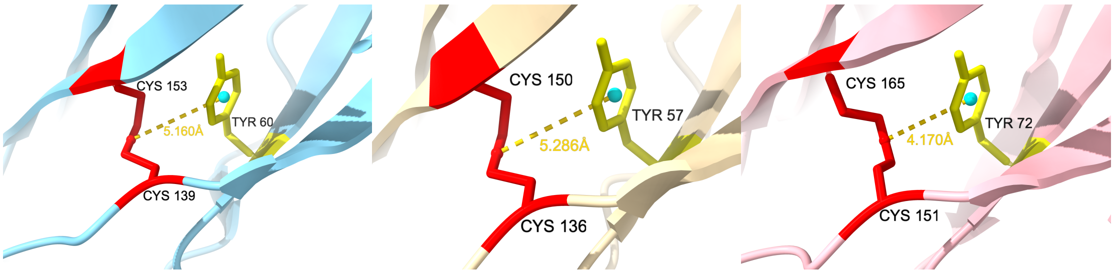

Supplementary Figure S5. Atomic distances between the Cys-loop disulfide bond and the packed tyrosine (Tyr) residue in each subunit of the eight RCSB PDB cryo-EM structures. Structures with the PDB IDs 6X3X, 6X3Z, 8DD2, 8DD3, 8G4N, 8SGO, 8VQY, and 8VRN **(a-i)** were examined with ChimeraX (Ver. 1.8) for the Tyr packed in the Cys-loop. The α1 subunit, β2 subunit, and γ2 subunit are shown in sky blue, yellow, and pink, respectively. The Tyr residues are labelled and depicted in yellow with a pseudo-atom in the middle of the aromatic ring for measurement of the distance. The distances are shown in yellow with a dotted line between the pseudo-atom and the disulfide bridge.

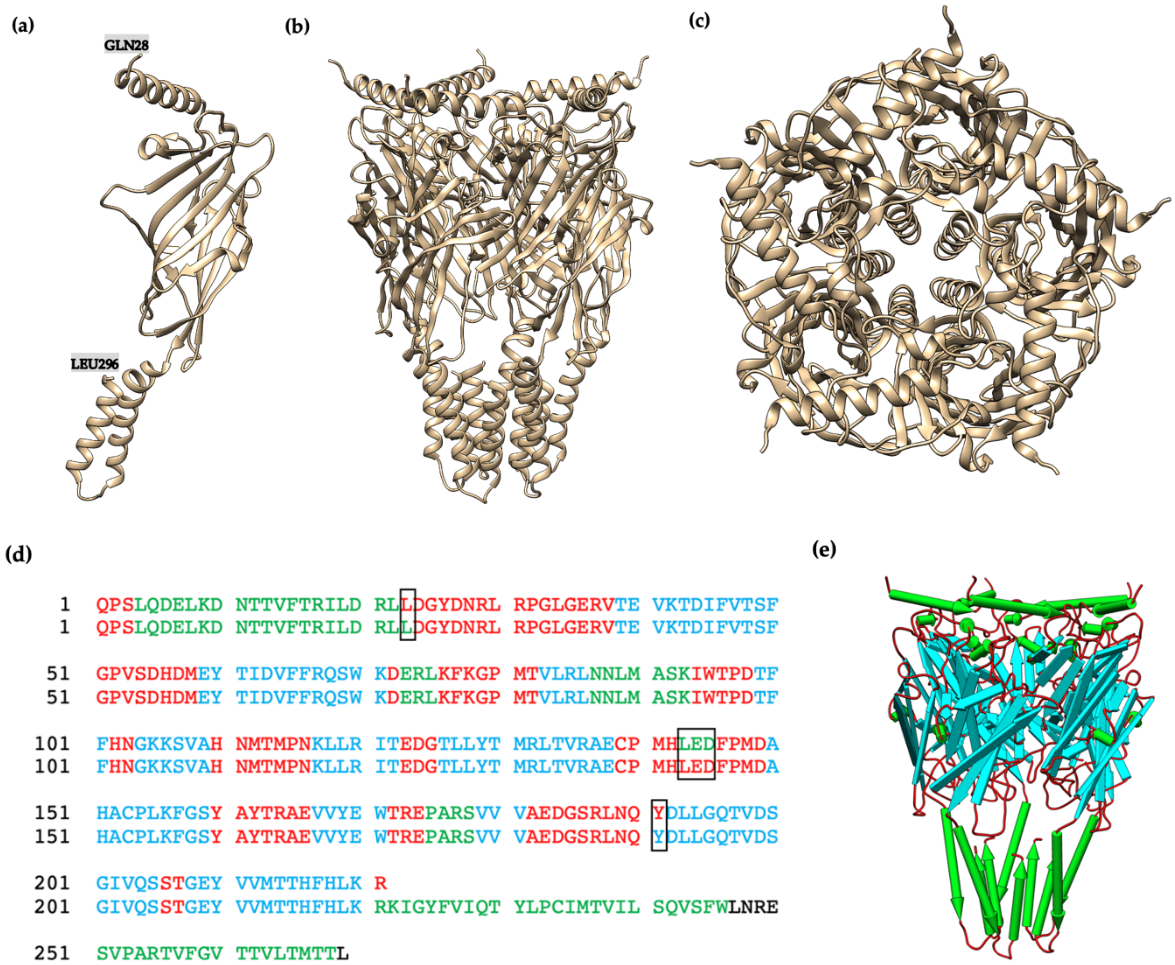

Supplementary Figure S6. α1 subunit Q28-L296 sequence and structure prediction from AlphaFold2. (a-c) AlphaFold2 prediction of the structure of the Q28-L296 fragment, with **(a)** single subunit, **(b)** side view, and **(c)** top view, as depicted in UCSF Chimera (Ver.1.17.3). **(d)** amino acid sequence colour-coded according to secondary structure matched with shorter Q28-R243 fragment expressed. Red = coils, green = alpha helices, blue = beta-strand. Differences are emphasised with a black square box, namely the extended alpha helix at L23 in the longer fragment, the extra alpha helix at L143-D154 for the shorter fragment, and lastly, the extended beta-sheet at Y191 for the longer fragment. **(e)** "Pipes-and-plank" model generated in Chimera according to secondary structure. Alpha helices are depicted as green "pipes", beta sheets as cyan "planks" and red strands as coils.

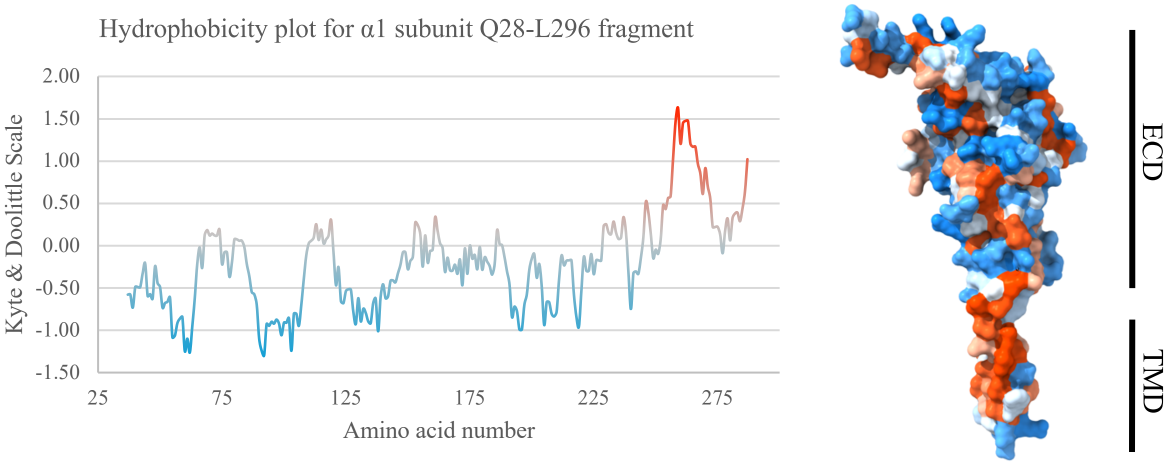

Supplementary Figure S7. Hydrophobicity of the *α*1 subunit Q28-L296 fragment. Left: the hydrophobicity plot according to the amino acid sequence of the subunit fragment, computed with window size 19 to identify hydrophobicity in the transmembrane domain. A negative score on the Kyle-Doolittle scale represents hydrophilicity and a positive score means the residue is relatively hydrophobic. Right: hydrophobicity surface depiction as portrayed by ChimeraX (Ver. 1.8). Orange = hydrophobic residues, white = neutral residues, and blue = hydrophilic residues. The extracellular domain (ECD) is relatively hydrophilic with more blue-coloured residues, and the transmembrane domain (TMD) is more hydrophobic with more orange-coloured residues.

```
β2   25   -------------------------QSVNDPSNMSLVKETVDRLLKGYDIRLRPDFGGPP   59
β3    1   MWGLAGGRLFGIFSAPVLVAVVCCAQSVNDPGNMSFVKETVDKLLKGYDIRLRPDFGGPP   60
                                   ******.***:******:*****************

β2        VAVGMNIDIASIDMVSEVNMDYTLTMYFQQAWRDKRLSYNVIPLNLTLDNRVADQLWVPD   119
β3        VCVGMNIDIASIDMVSEVNMDYTLTMYFQQYWRDKRLAYSGIPLNLTLDNRVADQLWVPD   120
          *.**************************** ******:*. *******************

β2        TYFLNDKKSFVHGVTVKNRMIRLHPDGTVLYGLRITTTAACMMDLRRYPLDEQNCTLEIE   179
β3        TYFLNDKKSFVHGVTVKNRMIRLHPDGTVLYGLRITTTAACMMDLRRYPLDEQNCTLEIE   180
          ************************************************************

β2        SYGYTTDDIEFYWRGDDNAVTGVTKIELPQFSIVDYKLITKKVVFSTGSYPRLSLSFKLK   239
β3        SYGYTTDDIEFYWRGGDKAVTGVERIELPQFSIVEHRLVSRNVVFATGAYPRLSLSFRLK   240
          ***************.*:***** :*********:::*::::***:**:********:**

β2        RNIG--------------------------------------------------------   243
β3        RNIGYFILQTYMPSILITILSWVSFWINYDASAARVALGITTVLTMTTINTHLRETLPKI   300
          ****
```

Supplementary Figure S8. Sequence alignment of the β2 Gln25-Gly243 fragment and the β3 subunit sequence. The percent identity matrix after MAFFT ClustalW alignment shows that the two sequences are 89.95% identical.
